
JOURNAL INFORMATION
==============================
NLM Title Abbreviation: Innov Surg Sci
Journal ID: Innov Surg Sci
NLM Journal ID: 101708165
Journal ID: iss

Innovative Surgical Sciences

EISSN: 2364-7485
Publisher: De Gruyter

ARTICLE INFORMATION
==============================
PMCID: PMC11005948
DOI: 10.1515/iss-2024-2005
Article ID: iss-2024-2005
Article version: 1
Subjects: Article

Abstracts DGKCH

Electronic publication date: 2024 Apr 9
Publication date: 2024 Apr
Volume: 9
Issue: Suppl1
First page: 209
Last page: 252
Copyright: © 2024 by Walter de Gruyter Berlin/Boston
Copyright year: 2024
Copyright holder: De Gruyter
License: This work is licensed under the Creative Commons Attribution 4.0 International License.
License URL: https://creativecommons.org/licenses/by/4.0/

Page count: 44

==============================

Abstracts – DCK 2024 – 141st Congress of the German Society of Surgery – Leipzig, April 24–26 • DOI 10.1515/iss-2024-2005
Innov Surg Sci 2024; 9 (Special Suppl 1): s209–s252

**Risk factors for the development of ischaemic colitis in patients with congenital heart defects**
ID: 69

Nariman Mokhaberi[1], Michalis Aftzoglou[1], Philipp Schneider[2], Daniel Biermann[2], Rainer Gerhard Kozlik-Feldmann[2], Christian Tomuschat[1]

[1]*Universitätsklinikum Hamburg-Eppendorf, Hamburg, Deutschland*, [2]*Universitäres Herz- und Gefäßzentrum Hamburg, Hamburg, Deutschland*

**Background:**
Necrotizing enterocolitis (NEC) is a common ailment in premature neonates, marked by intestinal inflammation, hypoxia, and eventual bowel necrosis. Its origins are multifaceted, stemming from intestinal immaturity, abnormal microbial colonization, and inadequate circulatory regulation, culminating in gut hyperinflammation. Despite advancements in preterm infant care, NEC continues to impose a significant burden of morbidity and mortality. Similarly, congenital heart disease (CHD), the most prevalent single-organ congenital anomaly, shares this burden of infant morbidity and mortality. Neonates with CHD face the risk of developing ischemic colitis (IC), with specific CHD phenotypes like ventricular septal defect, atrial septal defect, tetralogy of Fallot, univentricular heart, and coarctation of the aorta being prominent in Germany. Simple CHDs remain stable in incidence since 1996, while complex CHDs have seen a significant rise since 2008. In these CHD patients, compromised cardiac function leads to insufficiently oxygenated blood and reduced bowel wall blood flow, increasing the risk of bowel resection—a primary cause of short bowel syndrome in pediatric cases. Certain types of CHD, such as patent ductus arteriosus, significant left-to-right shunting, and atrioventricular canal defect, have been linked to NEC development, and even after surgery, ischemia and reperfusion injury remain potential threats. Notably, hypoplastic left heart syndrome, truncus arteriosus, and aortopulmonary window carry the highest NEC risk, particularly in premature or very low birth weight infants. Atrioventricular canal defects are a significant risk factor in this population. Our study aims to identify CHD patients who underwent surgery for ischemic colitis and evaluate the associated risk factors, enabling early detection and optimized care.

**Materials and methods:**
This retrospective study encompassed all patients with congenital heart defects (CHD) who underwent surgery for ischemic colitis at the Department of Pediatric Surgery, University Medical Center Hamburg-Eppendorf (UKE), from January 2013 to May 2023. The study adhered to the principles outlined in the Helsinki Declaration and received approval from the local institutional review board, the Hamburg Ethics Committee (2023-101012-BO-ff).

We collected and analyzed the following demographic and surgical data: age, gender, multiple gestation, birth weight, cardiac diagnosis, extracorporeal membrane oxygenation (ECMO) use, ECMO duration, date of colitis in relation to the cardiac procedure, number of infections prior to ischemic colitis, timing of cardiac surgery, timing of bowel surgery, bowel resection, stoma creation, enteral feeding, timing of patent ductus arteriosus (PDA) closure, and type of PDA closure (medical or surgical), as well as instances of mortality. Statistical analysis was carried out using Prism 9 for macOS (Version 9.5.0) and Microsoft Excel for Mac (Version 16.59).

**Results:**
This study included 35 patients, comprising 20 males and 15 females, among whom 20 were born prematurely, 10 had underlying syndromic diseases, and 9 were part of multiple pregnancies. The average birth weight was 2.11 kg, with one missing data point. ECMO treatment was administered to 4 out of the 34 patients, with an average duration of 2.23 days. Cardiac diagnoses were stratified based on congenital heart defect (CHD) complexity, resulting in 11 low-complex, 14 medium-complex, and 10 high-complex cases. Among these CHDs, 13 were cyanotic, and 22 were non-cyanotic. PDA closure was achieved through medical means in 7 patients and surgically in 8 others. After cardiac surgery, 25 patients underwent bowel surgery, 4 had bowel surgery before cardiac surgery, and 5 had bowel surgery

Open Access. © 2024 The Author(s), published by De Gruyter. This work is licensed under the Creative Commons Attribution 4.0 International License.

without cardiac surgery. Bowel resection was necessary for 25 patients, involving various types, including jejunal, ileal, right hemicolectomies, transverse colon resection, left hemicolectomies, and (sub)total colectomies. Apart from one patient, all 33 other patients required stoma formation. The median age at the time of bowel surgery was 41 days, spanning from 2 days to 10.07 years. The median interval between heart and bowel surgery was also 41 days. For bowel surgery, the median duration between the onset of abdominal symptoms and the surgical intervention was 1.24 days. A total of 10 enrolled patients did not survive, with 6 of these deaths directly associated with both heart and bowel surgeries.

**Conclusion:**
In this study, we analyzed 35 patients with diverse cardiac and bowel conditions, encompassing prematurity, syndromic diseases, and various congenital heart defects. Our results uncovered a significant mortality rate, notably attributed to the synergistic effects of heart and bowel surgeries. Notably, the rapid onset of abdominal symptoms following cardiac surgery should trigger suspicion of ischemia-reperfusion (I/R) injury, predisposing individuals to hyperinflammation, NEC, and subsequent bowel necrosis.

**The current state of surgical treatment for pediatric hydrocephalus in Germany**
ID: 231

Christine Drießler[1], Armin-Johannes Michel[2], Andrea Schmedding[3]

[1]*Klinikum Mutterhaus der Borromäerinnen gGmbH, Kinder- und Jugendchirurgie, Trier, Deutschland*, [2]*Landeskrankenhaus Salzburg - Universitätsklinikum der PMU, Universitätsklinik für Kinder- und Jugendchirurgie, Salzburg, Österreich*, [3]*Städtisches Klinikum Braunschweig, Kinderchirurgie und Kinderurologie, Braunschweig, Deutschland*

**Background:**
The ongoing revision of the AWMF S2k-Guideline “Kindlicher Hydrocephalus” prompted the conception of this study to capture the current medical care situation for hydrocephalus patients under the age of 18 in Germany.
Hydrocephalus is defined as “an active distension of the ventricular system resulting from inadequate passage of cerebrospinal fluid from its point of production within the cerebral ventricles to its point of absorption into the systemic circulation and is a common condition”.[1] It is a common condition, with infantile hydrocephalus having a prevalence of between one and 32 per 10,000 births.[2] The overall long-term outcome of patients with shunted pediatric hydrocephalus is poor and Gmeiner et al. emphasize the importance of life-long routine controls to avoid later complications.[3]
As a result, the transition of medical treatment from pediatric patients to adult patients is inevitable.
The objective is to investigate the current distribution of attending specialist departments that operate on pediatric hydrocephalus patients.

**Materials and methods:**
A survey will be conducted in pediatric surgical departments and neurosurgical departments in Germany using the online tool SoSci, an online questionnaire that allows single or multiple entries as well as open text input. The survey contains 17 questions and data will be collected anonymously. The final dataset will be analyzed descriptively.
Data dissemination will be carried out via the head physician mailing list, which is available to the board of the German Society of Pediatric Surgery (DGKCH). This study is in cooperation with “Arbeitskreis Spina bifida und Hydrocephalus” of DGKCH. To avoid potential redundancy, each department will be instructed to complete the questionnaire only once.

**Results:**
The survey is scheduled to be conducted over a period of 3 months, with anticipated dates November 2023 to January 2024.
The central research question focuses on how the operative treatment of children with hydrocephalus in Germany is split between pediatric surgeons and neurosurgeons. We intend to determine patient restrictions for each department consultant, for example age or weight.
Further issues include treatment type (operations, devices, endoscopy) and different etiologies of hydrocephalus that are offered at various departments.
This survey will reveal the cooperation structures of surgical departments with perinatal level 1 centers and shed light on the transition process from pediatric treatment to adult treatment.

**Conclusion:**
Our findings will provide insights into the current state of surgical treatment for pediatric hydrocephalus in Germany. In the final analysis, we need to determine treatment periods for the same patient cohort by two different specialities and where there is an overlap.
In all likelihood, there are existing cooperative structures but nationwide unification is worthwhile. Initial assessment of the treatment structures for this collective is required to identify potential improvements in easing the transition process and show the need for interdisciplinary cooperation.

**Literature**

1. Rekate HL. The definition and classification of hydrocephalus: a personal recommendation to stimulate debate. Cerebrospinal Fluid Res 2008;5:2.
2. Tully HM, Dobyns WB. Infantile hydrocephalus: a review of epidemiology, classification and causes. Eur J Med Genet 2014;57(8):359–68.
3. Gmeiner M, Wagner H, Schlögl C, et al. Adult Outcome in Shunted Pediatric Hydrocephalus: Long-Term Functional, Social, and Neurocognitive Results. World Neurosurg 2019;132:e314–23.

### STABILIZATION OF β-CATENIN INTERFERES WITH LIPOPOLYSACCHARIDE INDUCED P65 ACTIVATION ATTENUATING INFLAMMATORY SIGNALING IN INTESTINAL EPITHELIAL CELLS

ID: 233

Florentine Weise[1], Niklas Dressler[1], Nicole Peukert[1], Johannes Düß[1 2], Steffi Mayer[1], Martin Lacher[1], Jan Riedel[1]

[1]*University of Leipzig, Department of Pediatric Surgery, Leipzig, Deutschland*, [2]*John Hopkins University, Division of Pediatric Surgery and Department of Surgery, Baltimore, United States*

**Background:**

Impaired intestinal endotoxin tolerance is seen in human preterms suffering from necrotizing enterocolitis (NEC), but the underlying mechanism remains unknown. Activation of the TLR4/p65 pathway deregulates β-catenin abundance in intestinal epithelial cells (IECs) from human NEC patients. The cell adhesion molecule β-catenin plays a crucial role in transcriptional activation of proliferation and cell growth in intestinal epithelial cells (IECs). It has been shown that β-catenin acts either as a pro- or anti-inflammatory mediator in a cell and tissue specific behavior. This study aims to elucidate the effects of stabilized β-catenin upon lipopolysaccharide (LPS) induced inflammatory signaling in IECs.

**Materials and methods:**

Primary IECs were isolated from C57/BL6 WT mice or obtained as commercial available cell line (IEC-6, mIEC). To stabilize β-catenin, we treated IECs with the commercial GSK3 inhibitors CHIR99021 (10µM) or SB415286 (10, 25, 50µM) and stimulated these cells with LPS (0.1µg/mL). Post-translational modifications of p65 and β-catenin were assessed by Western Blot. Nuclear localisation was analyzed by Immunofluorescence or by nuclear protein extraction. Gene expression levels of pro-inflammatory mediators were determined via RT-PCR. Co-Immunoprecipitation was performed to evaluate p65-β-catenin protein interaction.

**Results:**

LPS signaling was associated with an increase in phosphorylation and nuclear accumulation of p65 along with a decrease of total β-catenin prone for proteosomal degradation (A). Inhibition of GSK3 stabilized β-catenin protein abundance in total and nuclear fractions which was associated with a nuclear retention of p65 (B). Gene expression of LPS induced inflammatory markers were attenuated upon β-catenin stabilization (C,D). This effect was consistent throughout all tested cell lines. We could not detect a direct protein-protein-interaction between p65 and β-catenin under different conditions of cultivation.

**Conclusion:**

Stabilization of β-catenin by inhibition of GSK3 attenuates LPS induced pro-inflammatory signaling by interfering with p65 activation. This protective effect assigns β-catenin as an anti-inflammatory mediator in intestinal inflammation with therapeutic potential.

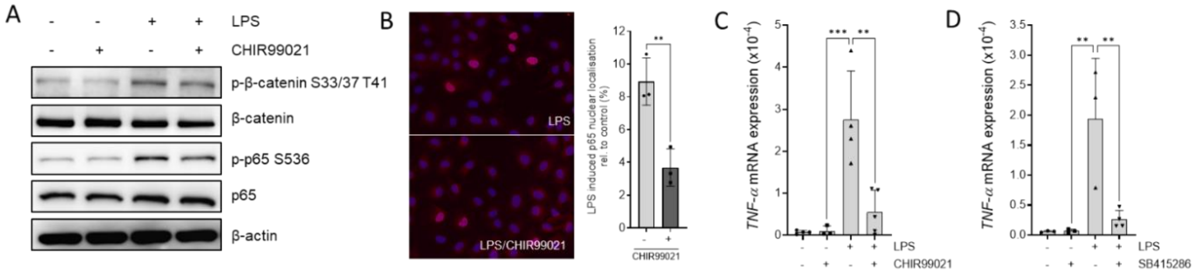

Figure 1: β-catenin stabilization by GSK3 inhibition impairs p65 activation and attenuates LPS induced pro-inflammatory gene expression (**p<0.01; ***p<0.001)

### STAT3 inhibition can improve lung budding in nitrofen induced lung hypoplasia

ID: 255

Paula Lieckfeldt[1 2], Jan Riedel[1], Moritz Markel[1 2], Florentine Dylong[1], Jaida Albrechtsen[2], Nicole Peukert[1], Martin Lacher[1], Jan-Hendrik Gosemann[1], Richard Keijzer[2], Richard Wagner[1 2]

[1]*Department of Pediatric Surgery, University of Leipzig, Leipzig, Deutschland*, [2]*Division of Pediatric Surgery, Pediatrics and Child Health and Physiology and Pathophysiology (Adjunct), University of Manitoba, Winnipeg, Canada*

**Background:**

Congenital diaphragmatic hernia (CDH) is a common birth defect associated with severe lung hypoplasia characterized by reduced airway branching and mesenchymal thickening, but the underlying molecular pathways are still poorly understood. Here we studied the transcriptional regulator STAT3 in nitrofen induced lung hypoplasia and the potential of STAT3 inhibition to restore lung budding.

**Materials and methods:**

For *in vivo* studies, we administered nitrofen (0.25mg/kg bodyweight) to pregnant rats at embryonic day 9 (E9) and harvested fetal lungs at E21 (n=3 per group). For *ex vivo* studies, we harvested fetal rat lungs at E14.5 and treated them with nitrofen (4.4mM), DMSO (vehicle) or STAT3 inhibitor (S3I-201 – 100µM) over 2-3 days in culture. Lung morphometric analysis (budding, growth, mesenchymal thickness) was performed by light microscopy daily (n=15-30 per group). Gene expression of STAT3 downstream target *Socs3* was analyzed by RT-PCR. Protein levels of total and phosphorylated STAT3 (pSTAT3 - Tyr705) were determined by Western Blot and Immunofluorescence.

**Results:**

STAT3 expression was significantly increased in the airway epithelium of *in vivo* nitrofen CDH lungs (E21). *Ex vivo* nitrofen treatment induced a hypoplastic phenotype indicated by smaller lung size (4.8±0.8 vs. 3.8±0.6 $mm^2$, p<0.001) and a reduced distal lung bud count (30±11 vs. 18±4, p<0.001). We detected an increased abundance of activated pSTAT3 in *ex vivo* hypoplastic lungs, which is in line with previous results *in vivo* (Piairo et al.). The status of activated STAT3 correlated with increased gene expression of the downstream target *Socs3*. STAT3 inhibition restored pSTAT3 levels and increased distal lung budding (18±4 vs. 27±5, p<0.001) and normalized peripheral mesenchymal thickness (0.08±0.02 vs. 0.11±0.02 mm, p<0.05).

**Conclusion:**

STAT3 is significantly increased in nitrofen CDH lungs *in vivo*. *Ex vivo* nitrofen treatment significantly activates STAT3 and induces a hypoplastic phenotype. STAT3 inhibition can partially rescue the hypoplastic phenotype *ex vivo,* but further studies to define the therapeutic potential of STAT3 inhibition in CDH are needed.

**RIP3-Caspase3-assay of Hirschsprung derived spheroid cultures revealed dysbalanced apoptosis upon proinflammatory stimulation**
ID: 259

Zhongwen LI[1], Clara Philippi[1], Johanna Hagens[1], Pauline Schuppert[1], Hans Schmidt[1], Laia Raluy[1], Magdalena Trochimiuk[1], Konrad Reinshagen[1], Christian Tomuschat[1]

[1]*Kinderchirurgie UKE, Hamburg, Deutschland*

**Background:**
Hirschsprung's disease (HD) is a prevalent congenital condition characterized by a failure of neural crest cells to populate the distal colon, leading to functional obstruction [1]. In a substantial subset of cases, HD is concomitant with Hirschsprung's disease-associated enterocolitis (HAEC), a severe inflammatory complication manifesting as symptoms such as fever, abdominal distention, and diarrhea [2]. Despite extensive research, the precise etiology of both HD and HAEC remains elusive. Recent findings have established a tentative association between HD and isolated instances of inflammatory bowel disease (IBD), although the underlying mechanisms remain poorly defined [3,4]. These recent investigations have implicated dysregulated apoptosis as a potential contributing factor in IBD [5]. This association has led us to postulate a similar apoptotic process occurring in HAEC. However, conventional methods exhibit limitations in their ability to accurately distinguish between various regulated cell death mechanisms. In contrast, the RIP3-Caspase3 assay represents a more nuanced approach for discerning complex cell death pathways, applicable to both living and deceased cells, thanks to its simplified antibody-based methodology [6]. Our research objectives revolve around investigating the hypothesis of imbalanced apoptosis in HD organoids, which have demonstrated the capability to replicate organ morphology in an in vitro setting [7]. Moreover, our approach will incorporate an innovative flow cytometry protocol, specifically developed for the purpose of studying cell death mechanisms within three-dimensional (3D) cultures.

**Materials and methods:**
After obtaining ethical approval (PV5251), our study involved in vitro modeling using HD organoids and control samples (n=10: 5 HD and 5 control organoids). To simulate an inflammatory response, we exposed these organoids to a cytokine cocktail consisting of TNF-α, IL-1beta and IL-6. To assess apoptosis, RIPK1-dependent apoptosis, necroptosis, and other cell death forms in organoid cells, we stained intracellular cell death proteins RIPK3 and Caspase-3, along with Zombie NIR staining, and quantified them using a new flow cytometry protocol for organoids. Sensitivity to stressors was compared between HD and CO organoids by measuring cell death levels under each culture and condition, with untreated samples serving as negative controls.

**Results:**
In our data, the overall comparison indicates increased cell death after acute cytokine treatment, as revealed by Zombie staining, in HD organoids. However, chronic exposure did not show significant differences. Notably, the percentage of Caspase-3+ cells among Zombie+ cells exceed 50%. Furthermore, the percentage of Caspase-3+ cells among Zombie- cells is smaller compared to Caspase-3+ cells among Zombie+ cells (Fig.1).

**Conclusion:**
HD-derived organoids are more susceptible to the cytokine cocktail compared to the control group. Our results reveal that HD-derived organoids are more susceptible to proinflammatory stimulation, showing increased apoptosis, compared to controls. This finding indicates a disrupted balance between cell survival and cell death in HD organoids and emphasizes an intrinsic proinflammatory status of the IECs of patients with HD. It also strengthens the observation of a link between HAEC and IBD. Also, our novel protocol promises to enhance the ability to dissect the intricacies of cell death pathways in the context of HAEC and provide valuable insights into its pathogenesis. This aligns with prior research and highlights the huge potential of patient-derived organoids and their relevance in future clinical treatment strategies and disease modelling.

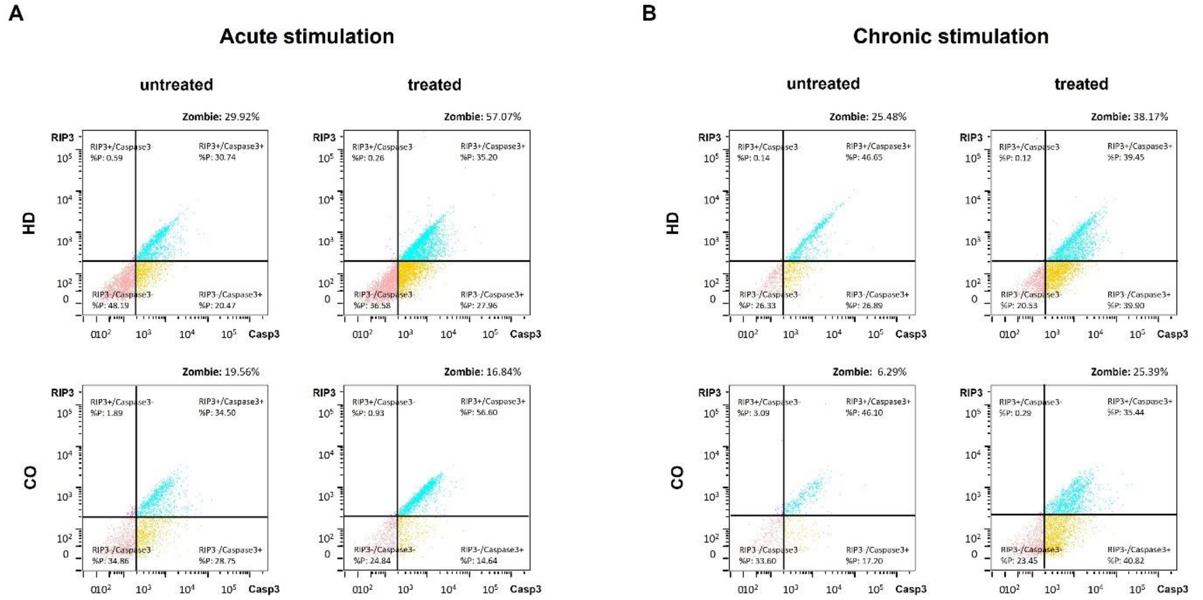

Fig. 1: Untreated cells or treated an acute (A) or chronic stimulation (B) setting as described were used to induce cell death and were labelled with fixable live cell stain Zombie NIR, Caspase-3-BV650 and RIP3-Alexa Fluor 488. After gating in live and dead cells were further gated for necroptosis, RIP1-dependent apoptosis and apoptosis respectively using a dot-plot of RIP3-PE vs Caspase-3-BV650 parameters. Necroptosis was described as an up-regulation of RIP3 in the RIP3-PE+ve/Caspase-3-BV650+ve population (MFI ± SEM, n = 3). RIP1-dependent apoptosis and apoptosis was described as the RIP3-PE+ve/Caspase-3-BV650+ve or RIP3-PE+ve/Caspase-3- BV650+ve population respectively.

references

1. Kyrklund, K. et al. ERNICA guidelines for the management of rectosigmoid Hirschsprung's disease. *Orphanet J Rare Dis* **15,** 164 (2020).
2. Gershon, E. M., Rodriguez, L. & Arbizu, R. A. Hirschsprung's disease associated enterocolitis: A comprehensive review. *World J Clin Pediatr* **12,** 68-76 (2023).
3. Granström, A.L.; Ludvigsson, J.F.; Wester, T. Clinical Characteristics and Validation of Diagnosis in Individuals with Hirschsprung Disease and Inflammatory Bowel Disease. J Pediatr Surg 2020, doi:10.1016/j.jpedsurg.2020.11.015.
4. Nakamura, H., Lim, T. & Puri, P. Inflammatory bowel disease in patients with Hirschsprung's disease: a systematic review and meta-analysis. *Pediatr Surg Int* **34,** 149-154 (2018).
5. Chen, Y. et al. Circulating exosomal microRNA-18a-5p accentuates intestinal inflammation in Hirschsprung-associated enterocolitis by targeting RORA. *Am J Transl Res* **13,** 4182-4196 (2021).
6. Lee, H.L.; Pike, R.; Chong, M.H.A.; Vossenkamper, A.; Warnes, G. Simultaneous Flow Cytometric Immunophenotyping of Necroptosis, Apoptosis and RIP1-Dependent Apoptosis. Methods 2018, 134–135, 56–66, doi:10.1016/j.ymeth.2017.10.013.
7. d'Aldebert, E. et al. Characterization of Human Colon Organoids From Inflammatory Bowel Disease Patients. *Front Cell Dev Biol* **8,** 363 (2020).

**Simulating the age-dependent susceptibility of intestinal epithelial cells to endotoxins - a murine in-vitro model for human NEC**
ID: 267

Thomas Wiemers[1], Jan Riedel[1], Niklas Dressler[1], Xiaoyan Feng[1], Nicole Peukert[1], Martin Lacher[1], Steffi Mayer[1]

[1]*Klinik und Poliklinik für Kinderchirurgie (UKL), Leipzig, Deutschland*

**Background:**
Exaggerated intestinal inflammation is associated with necrotizing enterocolitis (NEC) in preterm infants but the underlying mechanisms are poorly understood. Cell culture based models for NEC that reflect specific developmental stages and their characteristics are limited. To gain insides into cellular functionality we aimed to analyze the lipopolysaccharide (LPS) induced endotoxin response of murine intestinal epithelial cells at distinct intestinal developmental stages reflecting human intestinal maturation [1].

**Materials and methods:**
Primary intestinal epithelial cells (IECs) from small intestine samples of wild type C57BL/6J mice aged 1, 2, 4, and 8 weeks mimicking the intestinal maturation of fetal, preterm, term born as well as adult humans were isolated and stimulated with 1µg/mL LPS. Gene expression levels of markers for cellular maturation (*Si, Lyz*) and inflammation (*Tnf-α, IL-1β, Kc, IL-6, TLr-4, Md2*) were determined by RT-PCR. Phosphorylation status of p65 illustrating LPS mediated signaling was detected by Western Blot. Data were analyzed comparing the different age groups with and without LPS treatment at $p<0.05$.

**Results:**
Baseline gene expression of all maturation markers in IECs from 4- and 8-week old mice was significant higher compared to earlier ages. Contrariwise, baseline expression of all pro-inflammatory markers was significantly elevated in IECs from 1- and 2-week old animals compared to older ones. Basal *TLr-4* and *Md2* expression was highest in IECs from 2-week old mice. LPS treatment induced a strong increase in *Tnf-α* and *IL-1β at* early developmental stages (Fig.1A,B). LPS induced also a rapid phosphorylation of p65 30 minutes after stimulation in IECs from 2-week old mice. In 4-week old mice, this activation was diminished (Fig.1C).

**Conclusion:**
Immature stages of murine intestinal epithelial cells are characterized by a high degree of endotoxin susceptibility both, by a higher *basal expression* of pro-inflammatory mediators as well as a *stronger response* towards LPS. This developmentally impaired tolerance to LPS is also observed in NEC patients. The comparable functionality like endotoxin susceptibility for specific murine and human stages of intestinal development offer great potential to gain insights into molecular mechanisms that can be used as therapeutic targets for NEC in the future.

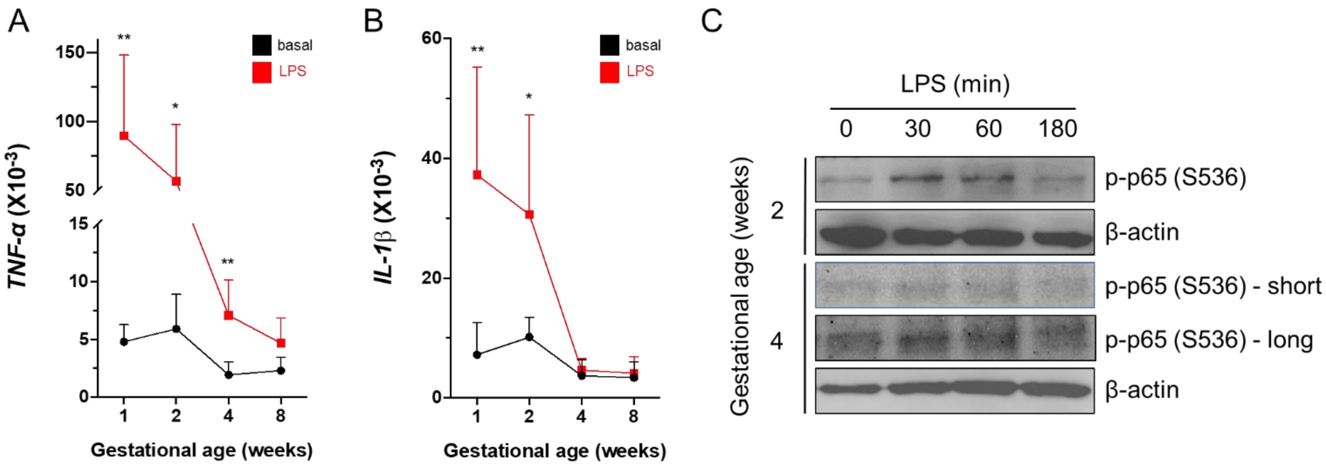

Figure 2: LPS induced inflammatory response at distinct gastrointestinal stages. Primary IECs were isolated and stimulated for 3 hours with 1µg/mL LPS. Pro-inflammatory gene expression of Tnf-α and IL-1β was determined by RT-PCR (A,B). Western Blot analysis of p65 (S536) from LPS (1µg/mL) treated IECs from 2- and 4-week old mice over time (C). *p<0.05; **p<0.01

**1.**Stanford AH, Gong H, Noonan M, et al. A direct comparison of mouse and human intestinal development using epithelial gene expression patterns. Pediatr Res 2020;88:66–76.

**Neutrophil Extracellular Traps mediate the formation of peritoneal adhesions**
ID: 326

Jasmin Knopf[1], Julia Elrod[2], Yuqing Lu[2], Janina Schoen[3], Leticija Zlatar[3], Martin Herrmann[2 3], Michael Boettcher[2]

[1]*Medizinische Fakultät Mannheim der Universität Heidelberg, Kinder- und Jugendchirurgie, Mannheim, Deutschland*, [1]*Medizinische Fakultät Mannheim der Universität Heidelberg, Kinder- und Jugendchirurgie, Mannheim, Deutschland*, [3]*Universitätsklinikum Erlangen, Medizin 3, Erlangen, Deutschland*

**Background:**
Peritoneal adhesions are highly prevalent conditions affecting 93-100% of patients following abdominal surgery displaying a major burden for patients and surgeons. They can cause serious complications such as intestinal obstruction and pelvic pain often requiring another surgery. Despite their high prevalence and their significant contribution to healthcare costs, their pathomechanism is still incompletely understood. Inflammation is commonly accepted as the most important element of peritoneal healing and the formation of adhesions. The innate immune system reacts within hours to clear pathogens and repair damaged tissues. Neutrophils are the predominant leukocyte of the innate immune system to be recruited to the site of injury and they can from neutrophil extracellular traps (NETs) upon contact with pathogens. NETs consist of a double-stranded DNA scaffold decorated with histones and neutrophil proteins. A delicate balance between NET formation and degradation is necessary to maintain this useful status of NETs and prevent the induction of hyperinflammation and the formation of immunothrombosis.

**Materials and methods:**
To analyze the effect of NETs on formation of peritoneal adhesions, we employed a laparotomy mouse model creating an injury using bipolar electrocoagulation. The formation of adhesion was assessed using two different adhesion scorings (Leach and Nair score) and by measuring the peritoneal thickness in histological stainings. The presence of NETs was detected by staining for extracellular DNA using the SYTOX$^{TM}$ Orange dye and additional immunofluorescence stainings for NET markers such as citrullinated histone H3 (citH3) and myeloperoxidase (MPO). To assess the influence of NET formation, we employed knockout (KO) mice for peptidyl arginine deiminase 4 (PAD4), displaying diminished NET formation. Since NET degradation is as important as NET formation, we also examined the formation of adhesions in KO mice for the two endogenous DNases DNase1 and DNase1l3. To transfer our findings to humans, we analyzed pediatric and adult adhesions obtained via surgery for NET formation by immunofluorescence and mass spectrometry. Since available treatments for the prevention of adhesion formation are scarce, we tested the effect of DNase1 as therapeutical option in our laparotomy mouse model.

**Results:**
Extracellular DNA accumulates in the adhesion 72h post injury confirming the involvement of NETs, whereas the Leach and Nair adhesion scores were highest after 21 days. This was further corroborated by immunofluorescence stainings for citH3 and MPO. Additionally, PAD4 KO mice (diminished NET formation) display significantly reduced adhesion formation as assessed by the adhesion scores and the measurement of peritoneal thickness. On the contrary, KO mice for the endogenous DNases DNase1 and DNase1l3 had significantly elevated formation of adhesions compared to wildtype animals. Interestingly, the absence of DNase1 not only lead to increased formation of adhesions but also influenced wound healing. In the human adhesion samples from both children and adults, the presence of NETs was confirmed by immunofluorescence staining for citH3 and MPO, as well as the presence of additional neutrophil proteins such as Cathepsin G in the mass spectrometry analyses. Of note, the mass spectrometry analyses also revealed the presence of modified fibrin peptides (such as citrullinated or oxidized fibrin). This indicates again the presence of NETs as reactive oxygen species and neutrophil enzymes such as PAD4 are potential drivers of these modifications. The modified fibrin peptides together with the absence of plasminogen or plasminogen activators also speaks for a prolonged persistence of fibrin potentially further stabilizing the adhesion. Lastly, the topical application of commercially available DNase1 during surgery and systemically 24 and 48h post surgery reduced the formation of adhesions significantly.

**Conclusion:**

Taken together, our work shows that NETs play an essential role in the formation of adhesions as a diminished NET formation (PAD4 KO mice) leads to reduced formation of peritoneal adhesions, whereas a reduced degradation of NETs (DNase1 and DNase1l3 KO mice) increases the formation of peritoneal adhesions significantly. The presence of NETs in adhesions was not only shown in our mouse model but also in human samples from surgery. Lastly, we showed that application of DNase1 is a promising therapeutic approach to prevent the formation of adhesions.

**Necrotizing enterocolitis and neutrophil extracellular traps: An organ in a dish**
ID: 330

Laura Blum[1], Jasmin Knopf[1], Julia Elrod[1], Deirdre Vincent[1], Michaela Klinke Petrowsky[1], Michael Boettcher[1]

[1]*Universitätsklinik Mannheim, Kinder-und Jugendchirurgie , Mannheim, Deutschland*

**Background:**
Necrotizing enterocolitis (NEC) is one of the most common causes of death affecting preterm infants. Despite intensive research, the pathogenesis is still not fully understood. However, it is known that the disease is a multifactorial process. In addition to a pathologic microbiome, it also includes an immature immune system. Therefore, one of the key factors is the uncontrolled, excessive immune response of the immature immune system, which specifically involves neutrophil recruitment. Neutrophil granulocytes play a special role in this process. As first-line responders, neutrophils respond via phagocytosis, building of reactive oxygen species (ROS), and formation of neutrophil extracellular traps (NETs); the latter being supposed to play a crucial role in the pathogenesis of NEC. NETs are net-like double-stranded DNA filaments and, upon pathogen contact, form a network of chromatin and proteins that can immobilize and thus eliminate bacteria, viruses, protozoa, or fungi. DNases are nucleases and are thus responsible for the degradation of DNA. Therefore, they can also degrade NETs. DNase 1 is one of the most commonly used, but DNase 1/3 turned out to be more effective. In comparison to DNase 1, it degrades not only DNA but also histones. Inhibition or degradation of NETs is likely to be an effective approach to reduce inflammation and intestinal damage. In mouse models, resolution of NETs using DNases has been shown to critically reduce the expression of NEC.

**Materials and methods:**
Intestinal tissue from healthy neonates and NEC tissue were used to generate organoids. Neutrophils and macrophages were isolated and co-cultured with the organoids. To compare the different immune reactions in healthy and NEC organoids, the co-cultures were stimulated with LPS and hypoxia was induced to simulate the most important risk factors for NEC. Immunofluorescence stainings, Flow Cytometry, ELISA and Western Blots were used to show the difference in NETs formation and in the pathomechanism. Organoid cultures were used to focus on different treatments, especially DNase 1. Next to this, the influence of macrophages as well as the interplay with neutrophils were examined, focusing on DNase1 and its potential to degrade NETs. Transwell experiments were used to investigate the neutrophil-epithelial barrier migration and to simulate neutrophil migration and their influence on apoptosis and necrosis.

**Results:**
Previous publications have clearly demonstrated NETs in intestinal tissue, serum, and stool of children with NEC, as well as in the context of NEC animal models. Inhibition of NETs is likely to be an effective approach to reduce inflammation and intestinal damage. In mouse models, resolution of NETs using DNases has been shown to critically reduce the expression of NEC. Current data also show that degradation of NETs can significantly reduce inflammation, intestinal cell apoptosis, and intestinal damage and the resulting NEC-related morbidity and mortality in animal models. We already demonstrated that there are differences between NEC and healthy organoids in their response to LPS treatment. NEC organoids showed increased apoptosis rates and TLR-4 expression. Next to this, the presence of NETs was also be shown by immunostaining. Different NETs markers were found in healthy organoids co-cultured with neutrophils and stimulated with LPS. LPS stimulates healthy co-cultures showed an increased NETs formation compared to unstimulated cultures, whereas NEC co-cultures have a higher neutrophil migration and NETs formation. This formation triggers apoptosis and necrosis of the epithelial barrier and increase the risk for sepsis and complications.
Macrophages have a positive effect against NETs formation in healthy and NEC organoids and showed decreased NETs formation compared to neutrophil co-cultures only.

**Conclusion:**
Our results suggest that neonates diagnosed with NEC may have congenital susceptibility due to increased TLR-4 expression. LPS resulted in an enhanced inflammatory response and NEC-like lesions in NEC organoids, particularly in the presence of neutrophils and NETs formation. Macrophages might have a positive effect against NETs formation and playing an important role in NEC pathogenesis. This "NEC in a Dish" model offers a promising alternative to animal experiments, but further research and adaptations are needed to fully replicate all aspects of NEC.

**Overactivated epithelial NF-κB disrupts lung development in congenital diaphragmatic hernia**
ID: 335

Florentine Dylong[1], Jan Riedel[1], Gaurang Amonkar[2], Nicole Peukert[1], Paula Lieckfeldt[1], Katinka Sturm[1], Benedikt Höxter[1], Wai Hei Tse[3], Yuichiro Miyake[3], Maria Moormann[1], Lena Bode[1], Steffi Mayer[1], Richard Keijzer[3], Martin Lacher[1], Xingbin Ai[2], Jan-Hendrik Gosemann[1], Richard Wagner[1 2]

[1]*University Hospital Leipzig, Department of Pediatric Surgery, Leipzig, Deutschland*, [2]*Massachusetts General Hospital, Division of Newborn Medicine, Department of Pediatrics, Boston, United States*, [3]*University of Manitoba and Children's Hospital Research Institute of Manitoba, Departments of Surgery, Pediatrics & Child Health, Physiology & Pathophysiology, Winnipeg, Canada*

**Background:**
CDH (congenital diaphragmatic hernia) with associated lung hypoplasia is a common developmental defect (1:2500) with significant neonatal mortality and morbidity which causes high expenses in our health system. Nevertheless, it is still characterized by a broadly undetermined pathobiology. Recent studies propose an enrichment of inflammatory processes in experimental and human CDH. Especially, the proinflammatory NF-κB pathway is repeatedly identified to be altered. The impact of its increased activation on lung development and the potential as a therapeutic target requires further investigation. In this study, we analyzed the activation status of the proinflammatory NF-κB pathway in CDH associated lung hypoplasia.

**Materials and methods:**
*Ex vivo* fetal lungs (E15) were cultured and exposed to nitrofen (4.4mM) for 48 hours or obtained *in vivo* from the nitrofen-rat model (E15, E21). Human fetal lung sections from CDH and non CDH patients were used from an established biobank. Dexamethasone (*ex vivo*: 1nM; *in vivo*: 0.25mg/kg bodyweight intraperitoneal) and curcumenol (10µM) were used as therapeutic treatment options. Lung morphometric analysis (budding, growth) was performed by bright field microscopy. Gene expression was analyzed by RT-PCR. Post-translational modifications of p65 (S536) or IκBα (S32) were determined by Western Blot analysis. Immunofluorescence from paraffin embedded sections was performed for p65 (S311).

**Results:**
We observed significantly impaired pseudoglandular airway branching (reduced lung budding and growth) in nitrofen-exposed lungs with hyperactivation of p65 in the airway epithelium at different developmental stages. Similar, human CDH samples indicated an elevated activation status of p65. NF-κB downstream targets like *Tnf-α*, *Cxcl1* and *Myc* showed increased mRNA levels in nitrofen exposed lungs. Treatment with the clinically established anti-inflammatory NF-κB antagonist dexamethasone partially rescued the phenotype of lung hypoplasia with recovered distal airway budding along with normalized NF-κB activity *ex vivo* and *in vivo*. Specific NF-κB inhibititon by curcumenol in fetal explants *ex vivo* resulted in a similar phenotype.

**Conclusion:**
Our results suggest an aberrant activation of NF-κB in human and nitrofen exposed hypoplastic (CDH) lungs. Pharmacological inhibition was able to restore abnormal lung characteristics indicating an underlying direct involvement of p65 within hypoplastic lung formation. Anti-inflammatory therapeutics in CDH associated lung hypoplasia can serve as potential strategies for further investigations.

**Disease entity and gestational age affect small intestinal microbiota of infants with enterotomies: a prospective two-center cohort study**
ID: 403

Tina Trautmann[1], Johanna Lichtenfeld[2], Nina Dietze[1], Corinna Bang[3], Janina Fuss[3], Andre Franke[3], Konrad Reinshagen[2], Michael Boettcher[1]

[1]*Kinder- und Jugendchirurgie , Universitätsmedizin Mannheim, Mannheim, Deutschland*, [2]*Klinik und Poliklinik für Kinderchirurgie, Universitätsklinikum Hamburg-Eppendorf, Hamburg*, [3]*Institut für Klinische Molekularbiologie (IKMB), Christian-Albrechts-Universität zu Kiel, Kiel, Deutschland*

**Background:**
Infants with proximal ostomies often suffer from severe fluid losses and electrolyte depletion, ultimately leading to a failure to thrive associated with poor long-term outcomes. An early-infantile microbial dysbiosis significantly aggravates weight faltering and has been associated with lifelong growth deficits and developmental impairment. Given that premature neonates with life-threatening bowel diseases are particularly susceptible, we thoroughly characterized the small intestinal microbiome for identification of specific targets to restore gut health.

**Materials and methods:**
A prospective two-center cohort study analyzed microbiome profiles of the ostomy effluent from 55 neonates with ileo- and/or jejunostoma based on gestational age (35 preterm *vs.* 20 term infants) and etiology (34 neonates with obstructive *vs.* 21 with inflammatory disease) from stoma-formation (TP1) until stoma reversal (TP7). Two representative samples from 29 patients were used to additionally correlate microbiota composition (16S sequencing) with function (metagenome analysis of DNA), and activity (metatranscriptome analysis of RNA).

**Results:**
Neonates and infants with proximal high-output ostomies showed significantly distinct microbial profiles based on their gestational age and etiology of disease ($p \leq 0.001$). Most strikingly, pre-term infants with inflammatory disease revealed microbial profiles associated with a severe dysbiosis that is highly susceptible for poor long-term outcomes. As expected, several therapy-related risk factors positively correlated with the extent of intestinal dysbiosis (i.e., long-term parenteral nutrition and antibiotic therapy).

**Conclusion:**
Profound perturbations of the intestinal microbiome by gestational age and disease etiology represent important risk-factors for poor long-term outcomes. Further studies should explore the effect of targeted interventions to counteract and improve microbial dysbiosis based on specific risk-profiles.

**Transcriptome analysis of intestinal colonic Hirschsprung organoids reveals intestinal barrier dysregulation under proinflammatory conditions.**
ID: 416

Johanna Hagens[1], Pauline Schuppert[1], Malik Alawi[2], Christian Müller[2], Konrad Reinshagen[1], Christian Tomuschat[1]

[1]*Universitätsklinikum Hamburg-Eppendorf, Klinik und Poliklinik für Kinderchirurgie, Hamburg, Deutschland*, [2]*Universitätsklinikum Hamburg-Eppendorf, Bioinformatik Facility, Hamburg, Deutschland*

**Background:**
The intestinal epithelial barrier is composed of three main groups of proteins: Tight junction proteins, such as ZO-1, Occludin and Claudins, along with adherens junctions represented by the cadherin family, serve as key regulators of paracellular diffusion and the dynamic structure of the epithelium [1-4]. To maintain structural stability under mechanical stress, Desmocollins and Desmogleins form Desmosomes [5, 6]. Transcriptome analysis is a novel technique that utilizes whole genome expression patterns to provide comprehensive insights into cellular regulatory processes in both physiological and pathological conditions [7, 8]. In our previous research, we investigated the intestinal barrier function in a large cohort of human Hirschsprung samples using intestinal colonic organoids. In this study, we aimed to reinforce our findings by integrating transcriptomic data.

**Materials and methods:**
We utilized intestinal colonic organoids derived from 10 human ganglionic Hirschsprung (gHIO) and 10 healthy control samples (CIO). These organoids were subjected to proinflammatory cytokine stimulation with human TNFα, IL-1β, and IL-6 to mimic intestinal inflammation. Acute stimulation was defined as a 72-hour treatment with daily medium changes while chronic stimulation extended to 96 hours with medium changes every two days. Untreated samples of both groups served as negative controls. Subsequently, organoids were pelleted and snap frozen at -80°C for whole mRNA expression analysis.

**Results:**
Transcriptome analysis revealed an overall reduced mRNA expression upon the genes of interest comparing acute with chronic stimulation, especially in gHIO samples. Acute stimulation led to a reduced expression of TJP1, OCLN and CLDN3 in gHIO while in CIO, only CLDN3 was negatively affected. CDH1 and DSC2 were elevated after stimulation in both gHIO and CIO. Chronic inflammation influenced TJP1 and OCLN negatively in both cohorts, while the other markers were nearly unaffected.

**Conclusion:**
Here, we introduce mRNA transcriptome data from a substantial cohort of Hirschsprung organoid samples exposed to proinflammatory conditions for the first time. Acute stimulation primarily impacted the tight junction proteins at the apical lateral membrane negatively, while reinforcing the expression of adherens junctions and desmosomes. In contrast, chronic inflammation induced milder damage and did not affect the basal compartment. These findings align with our prior observations, which demonstrated an apical-predominant destruction pattern of the intestinal barrier in immunofluorescence, along with an increased barrier permeability after acute compared to chronic stimulation, as indicated by FITC-Dextran diffusion. Consequently, harnessing transcriptomics for targeting gene expression levels in enteroids represents a promising avenue for unraveling the regulatory mechanisms underlying Hirschsprung Disease.

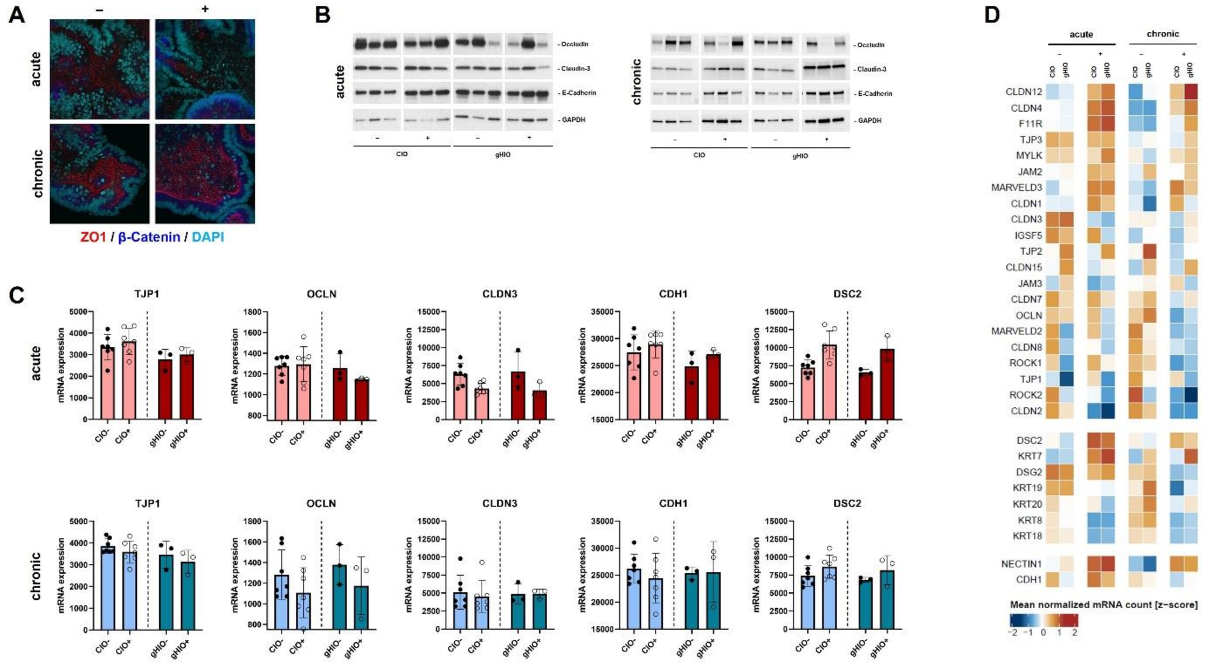

**Fig. 1:** Intestinal barrier marker impairment after acute and chronic proinflammatory stimulation was identified using different methods: **A** Confocal immunofluorescence staining of ZO-1 (red), β-Catenin (blue), and DAPI (cyan) highlights apical-predominant destruction after acute stimulation. **B** Altered protein expression of Occludin, Claudin-3 and E-Cadherin in Western Blot. **C** Transcriptome analysis reveals differences between tight junction (TJP1, OCLN, CLDN3), adherens junction (CDH1), and desmosome (DSC2) gene expressions before and after stimulation. **D** Heatmap of tight junction, adherens junction and desmosome gene expression patterns reveals alterations between gHIO and CIO enteroids before and after stimulation.

1. Schoultz, I. and V. Keita Å, The Intestinal Barrier and Current Techniques for the Assessment of Gut Permeability. Cells, 2020. 9(8).
2. Furuse, M., Introduction: claudins, tight junctions, and the paracellular barrier, in Current Topics in Membranes. 2010, Elsevier. p. 1-19.
3. Ivanov, A.I. and N.G. Naydenov, Dynamics and regulation of epithelial adherens junctions: recent discoveries and controversies. International review of cell and molecular biology, 2013. 303: p. 27-99.
4. Collinet, C. and T. Lecuit, Stability and dynamics of cell–cell junctions. Progress in molecular biology and translational science, 2013. 116: p. 25-47.
5. Kowalczyk, A.P. and K.J. Green, Structure, function, and regulation of desmosomes. Progress in molecular biology and translational science, 2013. 116: p. 95-118.
6. Joo, E.E. and K.M. Yamada, Cell Adhesion and movement, in Stem Cell Biology and Tissue Engineering in Dental Sciences. 2015, Elsevier. p. 61-72.
7. Stark, R., M. Grzelak, and J. Hadfield, RNA sequencing: the teenage years. Nat Rev Genet, 2019. 20(11): p. 631-656.
8. Yadav, D., et al., Overview and principles of bioengineering: the drivers of omics technologies, in Omics technologies and bio-engineering. 2018, Elsevier. p. 3-23.

Abstracts – DCK 2024 – 141st Congress of the German Society of Surgery – Leipzig, April 24–26 • DOI 10.1515/iss-2024-2005
Innov Surg Sci 2024; 9 (Special Suppl 1): s209–s252

**Neutrophil Extracellular Traps (NETs) in Post-Laparotomy Peritoneal Adhesions: Crosstalk, Adhesion Fibroblast Formation, and Therapeutic Potential of NET Inhibition**
ID: 492

Yuqing Lu[1], Julia Elrod[1]

[1]*University Medical Center Mannheim, Heidelberg University, Department of Pediatric Surgery, , Mannheim*

**Background:**
Peritoneal adhesions following surgery represent a significant clinical challenge. While these adhesions are frequently encountered, our understanding of their molecular genesis remains fragmentary. Recent studies have spotlighted fibroblast heterogeneity as a key element in various pathologies. Notably, such heterogeneity becomes more pronounced in the context of peritoneal adhesions. The term “adhesion fibroblast” describes a unique fibroblast phenotype that amalgamates characteristics of traditional fibroblasts and mesothelial cells.
Neutrophil extracellular traps (NETs) represent intricate structures composed of DNA, histones, and granule proteins extruded by neutrophils. A pivotal player in the NET formation process is Peptidyl arginine deiminase 4 (PAD4). It facilitates citrullination, where arginine residues on histones undergo transformation to citrulline. Drawing connections between NETs and adhesions, both are deeply entrenched in the body's tissue injury response and subsequent inflammation. Neutrophils swiftly mobilize to the injured sites as the first responders to tissue injuries. While their activation triggers NET release, primarily to thwart pathogens, the flip side sees NETs exacerbating tissue long-time damage due to their cytotoxic constituents.

**Materials and methods:**
Animal Experiments: We used a previously established adhesion model, inducing adhesions in the abdominal peritoneum via bipolar electrocoagulation for 2 seconds. For determining the optimal therapeutic approach, a series of treatment combinations were evaluated: Alvelestat: an inhibitor of neutrophil elastase (NE); BMP5: an inhibitor of Protein arginine deiminase 4 (PAD4); AZD5069: an inhibitor of C-X-C motif chemokine receptor 2 (CXCR2); NA11: an inhibitor of NADPH oxidase 2 (NOX-2); Pad2-in-1: an inhibitor of Protein arginine deiminase 2 (PAD2); Disulfiram: an inhibitor of Gasdermin D (GSDMD); Metformin: an inhibitor of High mobility group box 1(HMGB1); Colchicine: an inhibitor of NLR family pyrin domain containing 3 (NLPR3); DNase1; DNase1L3.
In Vitro Co-culture of Human Cells: Mesothelial cells and fibroblasts were isolated from the human peritoneum or adhesions utilizing a negative fluorescence-activated cell sorting (FACS) methodology. Neutrophils, harvested from human blood, were utilized to induce NETs with the help of PMA. After co-culturing NETs (with or without the treatment combinations) with fibroblast cells and mesothelial cells, Immunofluorescence, Western blotting, and PCR were used to probe both protein expression and transcriptomic alterations.
Tissue Specimen Analysis: Tissue specimens, sourced either from humans or mice, were subjected to co-staining after collection to investigate patterns of gene expression and the heterogeneity evident among abdominal adhesion fibroblasts obtained from both mouse and human samples. Additionally, co-staining localization and immunohistology test were performed for NETs markers and adhesion markers.

**Results:**
NETs were observed within post-surgical abdominal adhesions, and a salient feature of these adhesions is the heterogeneity among adhesion fibroblasts. Notably, these fibroblasts present phenotypic attributes reminiscent of both traditional fibroblasts and mesothelial cells. Cellular experiments revealed an intricate crosstalk facilitated by NETs, bridging the interaction between neutrophils and two primary cell types: fibroblasts and mesothelial cells. By inhibiting the NETs formation pathway, we observed a significant alleviation in adhesion levels within murine models. This amelioration can be attributed to a marked reduction in intercellular communication mediated by NETs.

**Conclusion:**

Our study underscores the critical role of NETs in peritoneal adhesion formation following abdominal surgeries. Post-laparotomy, neutrophils, through NETs, engage in intricate crosstalk with fibroblasts and mesothelium, leading to the rise of 'adhesion fibroblasts'. These fibroblasts play a central role in post-surgical peritoneal adhesions. Application of NET pathway inhibitors curtailed the communication between these cells, reducing activation and the subsequent development of adhesion fibroblasts. This insight paves the way for therapeutic interventions targeting postoperative adhesion formation.

**Inflammation and Neoangiogenesis after Exposure of Murine Lung Tissue to Neutrophil Extracellular Traps**
ID: 565

Richard Martel[1], Jasmin Knopf[1], Kian Samani[1], Michaela Klinke Petrowsky[1], Julia Elrod[1], Michael Boettcher[1]

[1]*Klinik für Kinder- und Jugendchirurgie, Universitätsklinikum Mannheim, Mannheim, Deutschland*

**Background:**
The systemic inflammation during ECMO therapy activates neutrophils in a way that triggers them to form Neutrophil Extracellular Traps (NETs). On the one hand, NETs play a key role in the formation of inflammatory thrombi, which account for a major part of the complications during ECMO in newborns. Therefore, the therapeutic inhibition of NET formation is a point of application to prevent embolic events during ECMO especially in newborns. On the other hand, NETs contain a proliferative effect in neoangiogenesis which is a desired process in the treatment of congenital malformations of the lung. To evaluate, to what extent neoangiogenesis is associated with NET induced inflammation, we studied the relationship between both in murine lung tissue.

**Materials and methods:**
Murine lungs in an explant culture were exposed to phorbol myristate acetate (PMA) activated neutrophils. Neoangiogenesis and inflammation was screened by a multiplex protein immunoassay (PEA). The topologic relationship between inflammation and neoangiogenesis was examined by immunofluorescence and quantified by digital image processing.

**Results:**
PEA showed a reliable increase of several cytokines, which was most expressed after exposure to activated neutrophils and less after exposure to neutrophils without explicit chemical activation. Vascular growth factors did not show an increased level in the protein assays. Immunofluorescence revealed a colocalization between areas with increased cytokine levels and signs of angiogenesis.

**Conclusion:**
Experimentally induced inflammation and angiogenesis are collocated when murine lung tissue is exposed to activated neutrophils. Therapeutic inhibition of NET formation may inhibit neoangiogenesis in lung tissue which needs to be considered when this novel therapeutic strategy is implemented.

### Follow-up outside the hospital setting – a pilot study for field testing of exercise capacity and motor skills in teenagers with esophageal atresia

ID: 620

Christine Driessler[1], David Friesen[2], Anke Widenmann-Grolig[3], Christine Joisten[2], Tatjana Tamara König[4]

[1]*Universitätsmedizin Mainz, Campus Trier, Klinikum Mutterhaus der Borromäerinnen Trier, Klinik für Kinderchirurgie, Trier, Deutschland*, [2]*Deutsche Sporthochschule Köln, Institut für Bewegungs- und Neurowissenschaft, Köln, Deutschland*, [3]*Selbsthilfeorganisation für Speiseröhrenerkrankungen KEKS e.V., Stuttgart, Deutschland*, [4]*Universitätsmedizin Mainz, Klinik und Poliklinik für Kinderchirurgie, Mainz, Deutschland*

**Background:**
Esophageal atresia (EA) is a rare malformation with relevant gastrointestinal and pulmonal co-morbidity, which requires life-long follow-up (1). Furthermore, EA is associated with impaired motor skills in childhood (2,3) and reduced exercise capacity in spiroergometry in adolescence (4-6). This test is time-consuming and costly. However, screening of exercise capacity in these patients is warranted, especially, since physical activity compared to healthy peers decreases with age (7). Many children with EA and other chronic conditions get lost to follow-up during adolescence, therefore data on this subgroup of EA patients only consists of small series (4, 6, 8). In order to increase adherence to the follow-up protocol and promote self-efficiency in these patients, simple tests for screening of exercise capacity and motor performance might be performed in a peer group outside the hospital setting. The Shuttle-Run-Test (SRT) is the gold standard for field-testing of exercise capacity and correlates with maximal oxygen uptake ($VO_2$ max) (9). The aim of this study was to evaluate the feasibility of the SRT for exercise capacity and Dordel-Koch-Test (DKT) for motor skills in youth with EA.

**Materials and methods:**
Adolescent members of the patient organisation KEKS e.V. with EA aged 12-16 years were invited to participate in field-testing of motor skills and exercise capacity at the German Sport University Cologne in April 2023. Patient history was taken, and participants were tested using the standardised and validated DKT and SRT. The DKT includes seven heterogenous items (lateral jumping, sit-and-reach, sit-ups, standing long jump, one-legged stand, push-ups and 6-minute-run), that are rated according to gender and age on a scale of 1-6 (consistent with the German school grading system, “1” being the most favourable outcome). Sufficient rest was provided between the 6-minute-run and SRT. For SRT, participants completed a 20m-shuttle-run at an increasing pace until exertion. The maximal speed and completed circuits define the test score (9). $VO_2$max was calculated according to Léger et al. (9) and rated according to a healthy reference cohort (10). Descriptive statistical analysis was performed.

**Results:**
In total, ten adolescents aged 12-16 years were included (mean age 13.9 years SD 1.2, 4 male, 6 female). Most patients were born with EA Gross Type C (n= 8) or Gross Type A (n=2). Three patients had persistent symptoms of tracheomalacia. The mean BMI-SDS was z= -0.66 (standard deviation 1.3). All participated in and finished all tests. Most symptoms (heartburn, nausea, respiratory symptoms) occurred during 6-minute-run (gastrointestinal symptoms in 3, dyspnea in 4 patients) and SRT (gastrointestinal symptoms in 4, respiratory symptoms in 6 patients). Two patients experienced bronchial obstruction with a need for bronchodilators after SRT. The mean scores according to gender and age were: lateral jumping 3.5 (standard deviation 0.8), sit-and-reach 3.7 (standard deviation 0.8), sit-ups 4.3 (standard deviation 1.0), standing long jump 3.4 (standard deviation 0.9), one-legged stand 2.2 (standard deviation 1.9), push-ups 4.9 (standard deviation 1.1) and 6-minute-run 3.7 (standard deviation 0.8). Mean percentile according to gender and age for $VO_2$max was 53.0 with a large variance (standard deviation 29.2, minimum 10$^{th}$ percentile, maximum 95$^{th}$ percentile).

**Conclusion:**

Abstracts – DCK 2024 – 141st Congress of the German Society of Surgery – Leipzig, April 24–26 • DOI 10.1515/iss-2024-2005
Innov Surg Sci 2024; 9 (Special Suppl 1): s209–s252

Even in our small cohort, exercise capacity and motor performance varied relevantly between individuals with esophageal atresia. The mean performance in most tests was close to average. Push-Ups and Sit-Ups yielded the lowest mean results. Most symptoms occurred during endurance tests. Notably, a certain degree of shortness of breath is a sign for successful completion of the test in general, since total exertion is the goal. Our data suggests that testing of greater cohorts than provided in the current literature is needed to produce representative data for all adolescents with EA. The DKT and SRT can be safely applied for field testing of EA patients yielding robust results. Testing in a "fun" environment in a group of peers outside the hospital setting might help improve adherence to follow-up, especially in this age group and might be applied to many chronic conditions.

1. Dingemann C, Eaton S, Aksnes G, Bagolan P, Cross KM, De Coppi P, et al. ERNICA Consensus Conference on the Management of Patients with Esophageal Atresia and Tracheoesophageal Fistula: Follow-up and Framework. Eur J Pediatr Surg. 2019.
2. König TT, Muensterer OJ. Physical Fitness and Locomotor Skills in Children With Esophageal Atresia-A Case Control Pilot Study. Frontiers in pediatrics. 2018;6:337.
3. Harmsen WJ, Aarsen FJ, van der Cammen-van Zijp MHM, van Rosmalen JM, Wijnen RMH, Tibboel D, et al. Developmental problems in patients with oesophageal atresia: a longitudinal follow-up study. Arch Dis Child Fetal Neonatal Ed. 2017;102(3):F214-f9.
4. Arneitz C, Windhaber J, Castellani C, Kienesberger B, Klymiuk I, Fasching G, et al. Cardiorespiratory performance capacity and airway microbiome in patients following primary repair of esophageal atresia. Pediatr Res. 2021;90(1):66-73.
5. Toussaint-Duyster LCC, van der Cammen-van Zijp MHM, Spoel M, Lam M, Wijnen RMH, de Jongste JC, et al. Determinants of exercise capacity in school-aged esophageal atresia patients. Pediatr Pulmonol. 2017;52(9):1198-205.
6. Dittrich R, Stock P, Rothe K, Degenhardt P. Pulmonary outcome of esophageal atresia patients and its potential causes in early childhood. J Pediatr Surg. 2017;52(8):1255-9.
7. König TT, Frankenbach ML, Gianicolo E, Holler AS, von Sochaczewski CO, Wessel L, et al. Habitual physical activity in patients born with oesophageal atresia: a multicenter cross-sectional study and comparison to a healthy reference cohort matched for gender and age. European journal of pediatrics. 2023.
8. Moinichen UI, Mikkelsen A, Faugli A, Morkrid L, H IJ, Emblem R. Impaired motor performance in adolescents with esophageal atresia. J Pediatr Surg. 2021;56(11):1926-31.
9. Léger LA, Mercier D, Gadoury C, Lambert J. The multistage 20 metre shuttle run test for aerobic fitness. J Sports Sci. 1988;6(2):93-101.
10. Tomkinson GR, Lang JJ, Tremblay MS, Dale M, LeBlanc AG, Belanger K, et al. International normative 20 m shuttle run values from 1 142 026 children and youth representing 50 countries. British journal of sports medicine. 2017;51(21):1545-54.

**Management of neonatal pediatric posthemorrhagic hydrocephalus (PHHC) in Germany – a claims data analysis**
ID: 712

Richard Gnatzy[1], Peter Zimmermann[1], Torben Schmidt[2], Stefan Bassler[3], Jona T. Stahmeyer[4], Martin Lacher[1], Matthias Krause[5], Jan Zeidler[2]

[1]*Department of Pediatric Surgery, University of Leipzig, Leipzig*, [2]*Center for Health Economics Research Hannover (CHERH), Leibniz University Hannover, Hannover*, [3]*AOK PLUS - Die Gesundheitskasse für Sachsen und Thüringen, Dresden*, [4]*AOK - Die Gesundheitskasse für Niedersachsen, Hannover*, [5]*Department of Neurosurgery, University of Leipzig, Leipzig*

**Background:**
To investigate the incidence, patient characteristics and current management of pediatric posthemorrhagic hydrocephalus (PHHC) in German pediatric (PD) and non-pediatric departments (non-PD).

**Materials and methods:**
In this observational cohort study, anonymized data were extracted from two large German statutory health insurances (~5.2 million clients) in a 7-year period (2010–2016). All patients with inpatient International Classification of Diseases (ICD) codes P52.- (intracranial non-traumatic hemorrhage of fetus and newborn) aged ≤ 12 months were included. Demographic, primary and consecutive clinical data were analyzed. The follow-up period was two years.

**Results:**
Of 1480 children with pediatric hydrocephalus (incidence 4.8/10.000 births), a total of 925 patients (63%) had an intraventricular hemorrhage. In 395 cases (42.7%), treatment was provided by a university/maximum care hospital (UM). A total of 103 patients (11.2%) underwent surgery for PHHC: in PD (n=83, 81.6%) and non-PD (n=14, 13.6%). Temporary and definite treatments were: Implantation of external ventricular drainage (EVD) (37.9%), Rickham reservoir (RES) (38.8%), ventriculoperitoneal shunt (VPS) (14.6%), and endoscopic third ventriculostomy (ETV) (8.7%). During the first inpatient treatment, complications occurred in 50 patients (49%) with at least one revisional surgery in 47 children (94%). After 24 months of follow-up 77 children (75%) finally received a VPS. All children admitted to UM were treated in PD (n=55) while 39.6% of the infants in Non-UM underwent surgery by neurosurgeons in non-PD (n=19). UM hospitals treated a higher number of preterms below $28^{th}$ gestation week (25.2% vs. 16.5%; p<0.001) compared to non-UM hospitals. At the same time the mortality rate of children treated at UM was significantly lower (4.2% vs. 7.3%; p=0.018).

**Conclusion:**
The surgical volume for PHHC in Germany is equally distributed between UM and non-UM. While the overall complication rate was very high (49% of children) the outcomes of children treated by pediatric surgeons and/or neurosurgeons were not different. Compared to UM hospitals non-UM hospitals treat a lower number of preterms below the $28^{th}$ gestation week and have a significantly higher mortality rate. Therefore, centralized care of these complex patients is mandatory.

**Superselective Embolisation for Partial and Total Splenectomy a new Method in the Management of Hypersplenism and Splenic Cysts in Children and Adolescents**
ID: 787

Salmai Turial[1], Maciej Pech[2], Volker Aumann[3], Alexandra Nyiredi[1]

[1]*University Medical Center Magdeburg, Department of Pediatric Surgery, Pediatric Traumatology and Pediatric Urology, Magdeburg, Deutschland*, [2]*University Medical Center Magdeburg, Department of Radiology and Nuclear Medicine, Magdeburg, Deutschland*, [3]*University Medical Center Magdeburg, Department of Pediatric Oncology und Hematology, Magdeburg, Deutschland*

**Background:**
Congenital spherocytosis, therapy resistant idiopathic thrombocytopenia purpura (TRITP) and large splenic cysts have a strong indication for a surgical partial or total splenectomy. The standard approaches for partial or total splenectomy comprise minimally invasive surgery (laparoscopic or robotic assisted surgery) and also open surgery. Conversely, the perioprative complications and costs make it a rather unpopular approach among patients, pediatricians, pediatric hematologists and also pediatric surgeons. Yet splenic volume reduction can also be achieved with the help of interventional radiology, using a superselective embolisation for partial and total splenectomy (SEPS, SETS). The purpose is to present our experience with superselective embolisation for partial and total splenectomy in a series of patients.

**Materials and methods:**
We reviewed our experience with SEPS and SETS in pediatric patients with hypersplenism or splenic cysts in between 2021-2023. A total of 12 patients were included. Five patients were diagnosed with spherocitosis five with sickle cell disease and two with a splenic cyst. All patients were referred to us for a partial or total splenectomy through the pediatric hematologist after they received the passive immunization according to the surgical splenectomy protocol prior to embolisation and a surgical team was always prepared for a surgical therapy. Monitoring was done by ultrasound on day one post-embolisation and by angio-CT on day two, hematological control on day three post-embolisation. Follow-up controls were conducted three and six months after the intervention.

**Results:**
SEPS comprised between 25-75% of the splenic volume and was documented with a angio-CT, this resulted in a reduction of the splenic volume in the follow-up check-ups. Three patients required re-embolisation because the initially embolised area was too small (ca 25%). In one case the embolisation resulted in intra-cystic bleeding that did not need a surgical intervention, but required radiological re-intervention. None of the patients required surgery. All patients had a good post-embolisation course with a hospital stay of fourteen to seven days. Initially all patients received an intravenous antibiotic prophylaxis for fourteen days. Due to the scarce side-effects reported, we reduced the hospital stay and intravenous antibiotics to one week. To note, is that none of the patients presented side-effects like: fever, pain or abdominal distension. Within a few days, platelet and leukocyte counts rose significantly in all patients that were maintained for a few weeks. Spleen size decreased significantly in all children. There were no infectious complications.

**Conclusion:**
According to our preliminary results superselective embolisation for partial and total splenectomy seems to be a safe and effective alternative to surgical partial or total splenectomy in the treatment of patients with hypersplenism and splenic cysts, being the method of choice in our department due to a high effectiveness, reduced costs and high patient compliancy.

Abstracts – DCK 2024 – 141st Congress of the German Society of Surgery – Leipzig, April 24–26 • DOI 10.1515/iss-2024-2005
Innov Surg Sci 2024; 9 (Special Suppl 1): s209–s252

**Electric cauterization of the lateral peritoneum overlying the internal inguinal ring reduces recurrence after laparoscopic extraperitoneal inguinal hernia repair in children**
ID: 546

Lena Bode[1], Jan-Hendrik Gosemann[1], Gabriel Götz[1], Steffi Mayer[1], Robin Wachowiak[1], Peter Zimmermann[1], Martin Lacher[1]

[1]*Uniklinikum Leipzig, Klinik und Poliklinik für Kinderchirurgie, Leipzig, Deutschland*

**Background:**
The objective of this study was to compare recurrence rates following laparoscopic extraperitoneal inguinal hernia repair in children with and without additional cauterization of the peritoneum overlying the internal inguinal ring.

**Materials and methods:**
Retrospective review of all children who underwent laparoscopic extraperitoneal repair for indirect inguinal hernia between 09/2015 and 08/2023 (8 years). All procedures were performed by trainees under supervision of five senior surgeons and video-recorded. Data included patient characteristics, surgical technique, operating time, conversion rate, the surgeon´s level of training, and postoperative complications including recurrence. In 12/2020 we modified our surgical technique by additional cauterization of the lateral peritoneum overlying the internal inguinal ring (from 8- to 2-o'clock on the left side and 10 to 4 o´clock on the right side) (spearing the peritoneum overlying the spermatic vessels and vas deferens). The recurrence rate of laparoscopic inguinal hernia repair without (non-cauterization group) and with cauterization of the lateral peritoneum (cauterization-group) were compared (Chi-Square analysis).

**Results:**
731 laparoscopic inguinal hernia repairs (371 unilateral, 180 bilateral) were performed in 551 children (410 boys, 141 girls; median age: 0.9 years (range 0 - 18.3 years); median weight: 7.9kg (range 2.0 – 102kg)). Median operative time without coagulation was 42 min (range 13 -133 min) for unilateral and 58 min (range 18-136 min) for bilateral herniorrhaphy. Refining the technique by coagulation lead to a median operative time of 36.5 min (range 15-98 min) for unilateral and 55min (range 24-104min) for bilateral herniorrhaphy. 442 hernias (60%) were treated with laparoscopic hernia repair without cauterization of the lateral peritoneum. Additional cauterization of the lateral peritoneum was performed in 289 hernia repairs (40%). In none of the patients, the vas deference or testicular vessels were injured. The recurrence rate in the non-cauterization group was significantly higher (17/442 (3.85%)) compared to the cauterization group (1/289 (0.35%) (p=0.0028).

**Conclusion:**
Electric cauterization of the lateral peritoneum overlying the internal inguinal ring reduces the recurrence rate of laparoscopic extraperitoneal inguinal hernia repair in children. Therefore, this technical modification has become our new standard.

**Pediatric Ovarian Hernia. Emergent, Urgent or Elective? Retrospective monoinstitutional cohort study**
ID: 704

Tomasz Baranski[1], Matthias Nissen[2], Ralf-Bodo Tröbs[3]

[1]*Helios Klinikum Duisburg, Klinik für Kinderchirurgie, Duisburg, Deutschland*, [2]*Marien Hospital Witten - Universitätsklinikum der Ruhr-Universität Bochum, Klinik für Kinderchirurgie, Witten, Deutschland*, [3]*KUNO Klinik St. Hedwig Krankenhaus Barmherzige Brüder, Regensburg, Klinik für Kinderchirurgie und Kinderorthopädie, Regensburg, Deutschland*

**Background:**
Sliding hernia of the ovary is the presenting feature in more than one fifth of female infantile inguinal hernia. Surgical reduction and herniorrhaphy is the definitive treatment. However, timing of surgery for ovarian hernia remains a matter of controversy. Two different strategies were discussed in literature. The aim of the presented study was to evaluate results and major in a single institution series.

**Materials and methods:**
This study encloses ninety-nine girls with inguinal hernia admitted over a three-year period (2015 to 2017). All children were underwent preoperative ultrasound and surgical hernia repair. We extracted epidemiologic and biometric data from the medical records an operation reports. During the observation period, we registered contralateral metachronous hernias and relapses. Patients were divided into two groups: prolapsed ovary at presentation (“prolapse”) vs. non-prolapse. Furthermore, we focused on a history of VLBW (very low birth weight) or gestational age below 35 weeks. Chi-squared test.

**Results:**
Thirty-eight children presented with prolapse of the ovary. Median age at surgery was 60 days for prolapse and 410 days for non-prolapse. The youngest infant with prolapse was 14 days old, and the youngest of the non-prolapse group 35 days. Thirty-six of 38 infants with prolapse were operated on urgently within 24 hours after admission (95%). The vast majority of non-prolapsed hernias was treated electively. One third of infants with prolapse (13 von 38, 34%) but only one tenth (8 of 61, 13%) of the non-prolapse group had a history of VLBW ($p < 0.001$). Two thirds (22 of 38, 58%) of the prolapse group had a gestational age below 35 weeks vs. one fifth (11 of 61, 18%) longer than 35 weeks ($p < 0.001$). Neither organ loss, nor metachronous hernia were registered. We had to re-operate one infant due to a bilateral relapse after laparoscopic surgery (1% relapse). There was no need for postoperative ventilation or blood transfusion.

**Conclusion:**
Our data show, that ovarian prolapse is a domain of VLBW infants and infants with gestational age below 35 weeks. Urgent operation in the case of prolapsed ovary was associated with preservation of the intact gonad in all cases. We conclude that herniorrhaphy within 24 hours is safe and effective.

Abstracts – DCK 2024 – 141st Congress of the German Society of Surgery – Leipzig, April 24–26 • DOI 10.1515/iss-2024-2005
Innov Surg Sci 2024; 9 (Special Suppl 1): s209–s252

**The minimally invasive correction of the inguinal hernia - a further development of the current technique**
ID: 815

Armin-Johannes Michel[1], Roman Metzger[1], Christoph Gebauer[1]

[1]*University Hospital Uniklinikum Salzburg, Clinic for Pediatric and Adolescent Surgery, Salzburg, Österreich*

**Background:**
Since the end of the 1990s, the technique of laparoscopic correction of pediatric inguinal hernia has been used worldwide. There are different surgical methods, mostly centre-specific, which differ both in the choice of trocar position or it's number and in the surgical procedure. We present the technique developed at the University Hospital Salzburg in the sense of an anatomically correct "real herniotomy" which is used equally for girls and boys. As not described before, we perform an absorbable reinforcement suture to attach the lower edge of the M. obliquus internus to the inguinal ligament in cases of wide open Processus vaginalis profundi. We present our case series of patients operated on from 2016 to 2022 (n=81) with evaluation of postoperative outcomes. The advantages and disadvantages compared to the conventional open procedure are discussed. With regard to the postoperative freedom from pain and mobilisation, the very good wound healing and the option to treat a contralateral open processus vaginalis in the same session, the laparoscopic herniotomy shows a benefit for the patient.

**Materials and methods:**
Patients are selected when the diagnosis is made. The choice of method is made after a detailed surgical explanation with the parents. The procedures are performed exclusively as a day surgery planned stay.
We present the technique of laparoscopic herniotomy on the basis of the anatomy, the suture technique and the use of the instruments. Accesses and trocars are 1x 5mm transumbilical and 2 x 3mm in the right and left mid-abdomen. A circumferential incision of the peritoneum at the level of the inguinalis profundus annulus is made, leaving out the deferent duct and the testicular vein or the rotundum ligament, followed by closure with a tabac pouch suture. In particularly large inguinal hernias, a reinforcing suture from the internal oblique muscle to the inguinal ligament is performed before peritoneal closure. In girls, the lig. rotundum is integrated into the suture. The methods are demonstrated with graphic illustrations and videos.

**Results:**
From 2016 to 2022, 81 patients (m/f = 42/39) underwent minimally invasive surgery for inguinal hernia at the University Department of Paediatric and Adolescent Surgery Salzburg. The mean age distribution is 7.1 years (1.4-15.7 years). Clinically, 37 patients showed a hernia on the right side, 20 on the left side and 7 on both sides. Intraoperatively, 17 times an open processus vaginalis on the contralateral side was diagnosed and closed at the same time. In this respect, 98 herniotomies were performed. In 27 patients with a particularly large hernia gap, the internal oblique muscle was fixed to the inguinal ligament. The mean operating time for unilateral hernia was 41 min, for bilateral hernia 57 min. No complications were recorded intra- or postoperatively. Follow-up showed one patient with a wound healing disorder at the umbilicus and one case with recurrence.

**Conclusion:**
We present a method for laparoscopic correction of inguinal hernia which can be used safely in both sexes and can be performed technically well with 5 or 3 mm trocars. Perioperative complications did not occur in our series. The operating time shortened rapidly as the learning curve progressed. With regard to the presentation of a clinically not yet conspicuous open processus vaginalis profundus, laparoscopy is the method of choice and, with one-stage treatment of both inguinal canals, it has a clear advantage over the open surgical technique.

Abstracts – DCK 2024 – 141st Congress of the German Society of Surgery – Leipzig, April 24–26 • DOI 10.1515/iss-2024-2005
Innov Surg Sci 2024; 9 (Special Suppl 1): s209–s252

**Renal artery occlusion after blunt abdominal trauma**
ID: 489

Friederike Heidtmann[1], Ilmi Alhussami[1], Matthias Waginger[2], Hans-Joachim Mentzel[2], Felicitas Eckoldt[1]

[1]*Klinik für Kinderchirurgie, Universitätsklinikum Jena, Jena, Deutschland*, [2]*Institut für Diagnostische und Interventionelle Radiologie - Sektion Kinderradiologie, Universitätsklinikum Jena, Jena, Deutschland*

**Background:**
Renal artery dissection is a rare entity that is challenging to diagnose. We present and discuss a case of blunt abdominal trauma leading to a dissection injury of the right renal artery.

**Materials and methods:**
**Case report**
A 14-year-old boy presented to the pediatric emergency department (ED) after blunt abdominal trauma the day before. He had taken a fall on his right side after jumping with his mountain bike. He had vomited two times. His physical examination showed an excoriation above his right iliac crest and on his right arm. Liver enzymes were elevated and there was microhematuria on urinalysis. Abdominal b-mode sonography in the ED showed no abdominal free fluid or other signs of injuries to the abdomen. The family was advised to admit the patient to the hospital for further observation but opted for discharge against medical advice. At a scheduled follow-up two days later, the patient reported no macrohematuria, no vomiting, but still some pain in his right abdomen. Ultrasound now showed a right perirenal hematoma as well as some retrovesical fluid. Liver enzymes were falling, while creatinine was slightly elevated at 102 µmol/l. The patient was again advised to remain at the hospital for observation but again opted for discharge against medical advice. He was advised therefore to keep bed rest and to emergently return to the ED in case of any worsening symptoms. At the next follow-up visit 8 days after trauma, he had returned to his normal activities and there was no more abdominal pain on exam, however, he had noticed slight macrohematuria. Ultrasound showed the right perirenal hematoma, while on duplex sonography, the flow of the right renal artery was reduced significantly at 1 cm distal to its aortic origin. Emergent MR-angiography revealed hyperintense material in the right renal artery, likely thrombotic material following a dissection lesion to the intima. The right kidney showed no sign of contrast uptake or excretion on MRI.
Percutaneous angiography and transluminal angioplasty (PTA) of the renal artery were performed emergently as a curative attempt. However, the right renal parenchyma continued to show no signs of perfusion after angioplasty. Due to the likely irreparable ischemic damage to the right kidney, stent implantation was not performed, as the risk for thrombotic events was deemed to outweigh the unlikely benefit of the stent. The patient was started on peri-interventional i.v. heparin and continued on low-molecular-heparin for 14 days. He continued to be stable and asked for discharge the day following the intervention. He continued to be monitored for global kidney function and the development of hypertension. One month after trauma, there was slight proteinuria, and GFR was mildly reduced with a Cys C of 1,28 mg/l. Six months after trauma, GFR was 78 ml/min*1,73m$^2$ (CAPA) and Cys C 1,11 mgl/l. After 12 months, GFR had returned to 97 ml/min*1,73 m$^2$ and Cys C was normal. The right kidney now showed atrophy on ultrasound, while the left kidney appeared to have compensated in size and function. He did not develop renovascular hypertension.
Notably, the patient presented again 2 years after the accident with ileofemoral deep vein thrombosis (DVT) with extension to the vena cava. He was successfully treated with an interventional thrombectomy. He was found to have low AT-III and is currently being evaluated for congenital AT-III deficiency, as his father and uncle were both diagnosed with the condition after DVT events.

**Results:**
Renal artery dissection is a rare entity that is challenging to diagnose. Blunt abdominal trauma can cause subintimal tears of the renal artery by a mechanism of sudden deceleration of the body (like in

Abstracts – DCK 2024 – 141st Congress of the German Society of Surgery – Leipzig, April 24–26 • DOI 10.1515/iss-2024-2005
Innov Surg Sci 2024; 9 (Special Suppl 1): s209–s252

motor vehicle accidents) or through contusion between the abdominal wall and spine. These tears may lead to subintimal dissection with thrombosis and consecutive stenotic lesions or total occlusion of the renal artery[1,2]. Patients with traumatic renal artery injury have mostly suffered significant trauma like falls or motor vehicle accidents, with most patients sustaining multiple injuries[2,3]. Patients can present with abdominal tenderness and/or hematuria, however, physical exam and urinalysis are usually not reliable for detection of renal artery injury[2,4]. MRI and CT angio are more sensitive for the detection of renal artery injury than ultrasound and are considered the diagnostic gold standard[4].
Renal artery dissections can heal spontaneously or with anticoagulation, however, some patients will develop arterial occlusion due to intramural hematoma, false lumen, or thrombotic infarcts[5]. In stable patients, medical management (anticoagulation and treatment for possible hypertension) is the preferred treatment for non-flow-limiting lesions[5]. Some patients will develop acute arterial occlusion with consecutive renal ischemia. Treatment options to restore arterial flow include endovascular (stenting) or surgical (bypass) options[5]. For partial renal artery occlusion, revascularization can be attempted with good outcomes even days after trauma[1,5]. If some arterial flow is preserved, renal artery stenting after stenosis due to traumatic arterial dissection seems to have no adverse long-term effects and can resolve or prevent renovascular hypertension, which otherwise makes eventual surgical nephrectomy necessary in a subset of patients[3-5].
Due to the vulnerability of the kidney to ischemic damage, total renal artery occlusions are difficult to treat successfully[5]. In complete renal artery occlusions, an ischemia time of less than 3 hours was associated with more positive outcomes[6]. Open surgical revascularisation is generally associated with poor results and a high risk for nephrectomy[6]. Endovascular treatment has been explored as an alternative, with mixed results[6]. Endovascular stenting >4 h after trauma is advised only if residual flow with preserved parenchymal perfusion is detected at angiography[5]. There remains a high risk for renal atrophy and hypertension even after initial successful stenting[6].

**Conclusion:**
In the case presented here, blunt abdominal trauma caused a traumatic lesion to the renal artery intima, resulting in ischemic damage to the right kidney. Due to the significant delay in presentation and diagnosis, the damage to the renal parenchyma was irreversible. The patient recovered global renal function by compensation of the left kidney. Whether renal function of the affected side can be preserved or not, patients should be closely monitored for the development of hypertension.

### Contemporary Preclinical Mouse Models for Pediatric Rhabdomyosarcoma: from Bedside to Bench to Bedside

ID: 214

**Kategorie: DGKCH - Onkologie (Sarkome)**

Illya Martynov[1], Lajwanti Dhaka[1], Paul Hoyer[1], Mohammad Vahdad[1], Guido Seitz[1]

[1]*Department of Pediatric Surgery and Urology, University Hospital Giessen-Marburg*

**Background:**
Rhabdomyosarcoma (RMS) is the most common pediatric soft-tissue malignancy, characterized by high clinicalopathological and molecular heterogeneity. Preclinical in vivo models are essential for advancing our understanding of RMS oncobiology and developing novel treatment strategies. However, the diversity of scholarly data on preclinical RMS studies may challenge scientists and clinicians. Hence, we performed a systematic literature survey of contemporary RMS mouse models to characterize their phenotypes and assess the translational relevance

**Materials and methods:**
We identified articles published between 01/08/2018 and 01/08/2023 by searching PubMed and Web of Science databases.

**Results:**
Out of 713 records screened, 118 studies (26.9%) were included in the qualitative synthesis. Cell line-derived xenografts were the most commonly utilized (63.6%), followed by patient-derived xenografts and syngeneic models (11.9% each), and genetically engineered mouse models (GEEM) (5.9%). Combinations of different model categories were reported in 5.9% of studies. One study employed a virus-induced RMS model. Overall, 40.0% of studies established alveolar RMS, while 38.7% embryonal phenotypes. There were 20.0% of studies that involved a combination of both aRMS and eRMS subtypes. In one study (1.3%) the RMS phenotype was spindle cell/sclerosing. Subcutaneous xenografts (55.9%) were more frequently used compared to orthotopic models (24.6%). Notably, all the employed cell lines to establish CDX did not derive from primary untreated tumors. Only the minority of studies investigated disseminated RMS phenotypes (13.6%). The utilization areas of RMS models included testing novel drugs (54.2%), studying tumorigenesis (47.5%), tumor modeling (16.1%), imaging (7.6%), radiotherapy (5.1%), long-term effects related to radiotherapy (2.5%), and investigating biomarkers (0.8%). Notably, there were no preclinical studies focusing on surgery.

**Conclusion:**
This study provides an up-to-date review of contemporary preclinical studies on RMS, highlighting the need of mouse models with dissemination phenotypes and cell lines from primary untreated tumors. Furthemroe, efforts should be directed towards underexplored areas such as surgery, radiotherapy, long-term effects, and biomarkers.

**Importance of Adequate Surgical Local Control in Paratesticular Rhabdomyosarcoma: Data From the ”Cooperative Weichteilsarkom Studiengruppe”Trials CWS-96, 2002P, and European Soft Tissue Sarcoma Registry (SoTiSaR)**
ID: 234

Illya Martynov[1], Monika Sparber-Sauer[2], Amadeus Heinz[3], Christian Vokuhl[4], Martin Ebinger[3], Jens Gesche[5], Marc Münter[6], Ewa Koscielniak[2], Jörg Fuchs[7], Guido Seitz[1]

[1]*Department of Pediatric Surgery, University Hospital Giessen-Marburg, Marburg, Germany*, [2]*Klinikum der Landeshauptstadt Stuttgart gKAöR, Olgahospital, Stuttgart Cancer Center, Zentrum für Kinder-, Jugend- und Frauenmedizin, Pädiatrie 5 (Pädiatrische Onkologie, Hämatologie, Immunologie), Stuttgart, Germany*, [3]*Department of Pediatric Hematology and Oncology, University Children´s Hospital, Tuebingen, Germany*, [4]*Section of Pediatric Pathology, Department of Pathology, Bonn, Germany*, [5]*Josefinum KJF Klinik, Augsburg, Deutschland*, [6]*Department of Radiooncology, Klinikum Stuttgart, Germany*, [7]*Department of Pediatric Surgery and Urology, University Children's Hospital, Tuebingen, Germany*

**Background:**
Attaining local control through adequately executed surgery leads to an excellent prognosis for patients with localized paratesticular rhabdomyosarcoma (PTRMS). However, an inappropriate initial diagnosis and surgery will necessitate additional treatment to achieve desired outcomes. We aimed to assess how the quality of the initial and subsequent local surgical control impact survival.

**Materials and methods:**
Overall, n=241 patients with localized (IRS I-III) and metastatic (IRS IV) PTRMS were enrolled in the three Cooperative Weichteilsarkom Studiengruppe (CWS) trials (CWS-96, 2002P) and European Soft Tissue Sarcoma Registry (SoTiSaR). A total of n=196 patients (IRS I-IV) were eligible for this analysis. All patients were treated according the respective protocols with resection, chemotherapy (CHT) and radiotherapy (RT) if indicated. The 5-year overall survival (OS) and the 5-year event-free survival (EFS) were calculated using Kaplan-Meier estimates.

**Results:**
Among 196 patients (all fusion gene-negative RMS, median age 8.4 years, median follow up 5.7 years), 106 (54.1%) had initial R0 resection (semicastration [93.4%]; hemiscrotectomy [1.9%], scrotal tumor resection [4.7%]), 67 (34.2%) patients had pretreatment re-excision (PRE). All patients were treated with CHT and 21 (10.7%) with RT of lymph nodes. The 5-year OS and EFS for overall study cohort (IRS I-IV) was 90.1% and 84.1%, respectively. For patients with localized PTRMS (n=183), the 5-year OS was 94.0% and the 5-year EFS 87.3%. Overall, protocol violations during first surgery (PV-FS) were observed in 42% of patients. The 5-year OS and EFS for overall study cohort (IRS I-IV) was 90.1% and 84.1%, respectively. For patients with localized PTRMS (n=183), the 5-year OS was 94.0% and the 5-year EFS 87.3%. Overall, protocol violations during first surgery (PV-FS) were observed in 42% of patients. This resulted in higher rates of R1/R2 resections (76% vs. 22%, p<0.001) with a need for pretreatment re-excision (PRE) surgeries (69% vs. 10%, p<0.001) compared to patients undergoing correct primary surgery. Although PV-FS did not influence the 5-years OS and EFS in localized (p=0.312; p=0.581) and overall PTRMS cohorts (p=0.360; p=0.574), log-rang test revealed that R0 status after PRE was a significant prognostic factor for 5-year OS in both localized (R1 vs. R0; 77.8% [54.9%-100%] vs. 97.5% [92.8%-100%], p=0.018) and overall (R1 vs. R0; 70% [46.7%-100%] vs. 97.6% [93%-100%], p=0.002) PTRMS cohorts.

**Conclusion:**
PV-FS is associated with high rates of initial incomplete resections and a need for PRE to maintain excellent OS and EFS. Achieving R0 status after PRE through re-excision of tumor margins, semicastration or hemiscrotectomy is the most important surgical factor affecting 5-years OS for patient with both localized and metastasized PTRMS.

**Pediatric retroperitoneal masses presenting with acute abdomen - initial assessment and management**
ID: 526

Friederike Heidtmann[1], Christina Campbell[1 1], Ilmi Alhussami[1], Hans-Joachim Mentzel[2], Felicitas Eckoldt[1]

[1]*Klinik für Kinderchirurgie, Universitätsklinikum Jena, Jena*, [2]*Institut für Diagnostische und Interventionelle Radiologie - Sektion Kinderradiologie, Universitätsklinikum Jena, Jena, Deutschland*

**Background:**
When diagnosing a child or adolescent with acute abdominal pain, the physician will consider likely causes like obstruction, ischemia, inflammation, or trauma/bleeding. Pediatric retroperitoneal tumors can sometimes present as acute abdomen in the case of subcapsular hemorrhage, tumor rupture, or necrosis. Hemorrhage of previously undetected retroperitoneal tumors led to the diagnosis in the two cases described here.
We demonstrate the need for proper initial assessment – including appropriate imaging[1] – determined by careful history and clinical examination. Both cases also offer successful illustrations of stabilization by non-surgical measures and delayed, non-emergent resection preserving a high standard of surgical-oncological principle.

**Materials and methods:**
**Case Nr. 1**
A four-year-old girl was transferred by a secondary care hospital because of acute abdominal pain, vomiting, and abnormal findings on ultrasound. The patient began to show signs of hemodynamic instability and respiratory failure as well as a distended, tender abdomen and a large, palpable left-sided mass – confirmed by immediate sonography at our institution. Given the increasing general instability of the patient and previous findings, an additional abdominal contrast-enhanced CT scan was performed emergently, confirming the diagnosis of a ruptured nephroblastoma of the left kidney and retroperitoneal hemorrhage – however, active bleeding could be ruled out; free fluids intraperitoneally were encountered via sonography two days later.
No major or related previous illnesses were reported; however, about three weeks prior the girl had jumped out of a car driving at a slow speed (12 km/h). Initial work-up in the hospital of referral following the accident had shown a hematoma of the medial thighs proximally; imaging of any kind had not been performed at this time.
Fluid resuscitation and transfusion of 250 ml of blood stabilized the patient in the intensive care unit (ICU); further work-up (abdominal MRI, thoracic CT) was then completed, and neoadjuvant chemotherapy initiated (SIOP-RTSG 2016, Doxorubicin, Actinomycin D, Vincristin). Following protocol, a tumor nephrectomy was performed after four weeks successfully. Currently, the patient is undergoing adjuvant chemotherapy and abdominal radiation.

**Case Nr. 2**
A seven-year-old previously healthy girl presented to an outside hospital with an acute onset of pain in the right abdomen and renal angle. She had vomited twice and appeared somnolent. There was no fever, dysuria, or recent trauma. Abdominal ultrasound showed an inhomogeneous mass above the right kidney. Clinically, virilization was notable. The patient was urgently referred to our emergency department (ED) and admitted for further diagnostics. Upon arrival, vitals were stable and Hb was normal (7.1 mmol/l). However, the patient continued to vomit, was increasingly obtunded, and developed tachycardia in the following hours. Hb dropped to 4.6 mmol/l. She was admitted to the pediatric ICU for hemorrhagic shock, where she stabilized after a bolus of IV fluids, a transfusion of 250 ml of blood, and tranexamic acid.
Emergency contrast-enhanced CT showed a hemorrhagic suprarenal mass and consecutive retroperitoneal hematoma; major vascular injury requiring acute surgical intervention could, however, thereby be dismissed. The patient developed free abdominal fluid as well as a bloody right pleural effusion but continued to be stable. After completion of imaging and endocrinologic work-up, which was suggestive of adrenal cortex carcinoma, the tumor was therefore resected by laparotomy. Intraoperatively, the mass presented as a large hematoma combined with necrotic tissue contained retroperitoneally, displacing, but not infiltrating renal parenchyma or vessels. Tumor tissue and hematoma were not differentiated clearly due to the previous hemorrhagic event. Visually complete removal of the hematoma and remaining tissue was achieved sparing the right kidney. The patient is recovering well, while histologic typing is pending.

**Results:**

Retroperitoneal malignant tumors are common pediatric tumors, with neuroblastoma and nephroblastoma being the most frequent. On occasion, abdominal trauma can lead to the diagnosis of an abdominal mass[2]. Abdominal ultrasound is a valuable and readily available diagnostic tool to identify abdominal masses in the ED and to guide further diagnostics and treatment. MRI studies are usually needed for further confirmation and differentiation. CT is the method of choice in emergency situations[3]. A retrospective epidemiologic study found an incidence of cancer in the pediatric ED of 22.8 cases per 100,000 ED visits. The most prevalent diagnoses were cancers of the hematologic system (37.7%), followed by the central nervous system (31.2%) and the abdomen (22.1%)[4]. Another retrospective single-center study found that out of 156 patients who presented to the ED with abdominal pain and received CT imaging of the abdomen, five were diagnosed with intraabdominal masses. All five masses were initially detected via ultrasound[5].

Nephroblastomas often present as an abdominal mass diagnosed incidentally during routine checkups but can present more acutely with abdominal pain when tumor rupture or subcapsular hemorrhage occurs[6]. Tumor rupture was not associated with reduced overall long-term survival compared to non-ruptured stage III Wilms tumor; however, the risk of metastasis and recurrence is increased[7]. Primary resection of ruptured tumors is not advised following SIOP-Protocol; especially if the tumor is large and crosses the midline, when there is a Cava-embolus or extensive, non-localized rupture[7].

Adrenal tumors apart from neuroblastoma are rare in children. They can present clinically with signs of excess hormone production but are often identified at a late stage with poor prognosis. Radical open adrenalectomy is the treatment of choice for Adrenocortical Carcinomas[8]. Few reports of ruptured adrenocortical tumors in children are available; emergent adrenalectomy became necessary in most of the reported cases, whilst one patient was stabilized by trans-arterial embolization[9].

**Conclusion:**

We conclude that the occurrence of pediatric cancer in the ED, especially on a referral hospital level, may be higher than the incidence of these tumors in the general population suggests[10]. The diagnostic approach to abdominal complaints in children presenting to the ED should consider underlying tumors in the differential[4]. Ultrasound is an effective tool for the evaluation for intraabdominal masses in children in the ED[11,12]. If these patients present with acute, emergent symptoms and even acute hemorrhage, non-surgical initial stabilization and adequately delayed resection after careful work-up are preferred whenever possible.

**Approaching peritoneal tumour dissemination in children and young adults with cytoreductive surgery and hyperthermic intraperitoneal chemotherapy: a two-centre experience and review of the literature**
ID: 560

Sara Peggion[1], Christiane Staude[2], Wolfram Trudo Knoefel[2], Konrad Reinshagen[1], Stefan Klohs[1], Wilhelm Wößmann[3], Rüdiger Wessalowski[4]

[1]*University Medical Center Hamburg-Eppendorf, Department of Pediatric Surgery, Hamburg, Deutschland*, [2]*Düsseldorf University Hospital, Department of General, Visceral and Pediatric Surgery, Düsseldorf*, [3]*University Medical Center Hamburg-Eppendorf, Department of Pediatric Hematology and Oncology, Hamburg, Deutschland*, [4]*Düsseldorf University Hospital, Department of Pediatric Oncology, Hematology and Immunology, Düsseldorf, Deutschland*

**Background:**
Peritoneal dissemination in case of solid abdominal malignant tumours in children and young adults is a rare condition associated with poor prognosis. [1] In patients fulfilling well defined criteria cytoreductive surgery (CRS) followed by hyperthermic intraperitoneal chemotherapy (HIPEC) can be performed within a multimodal therapy strategy. Although CRS has been standardized (Sugarbaker et al.), applied HIPEC protocols remain heterogenous. [2] Surgical radicality is mandatory to achieve sufficient drug penetration, since this is limited to few cell layers. [3] On the other hand this explains why multiple studies showed this approach to be considered safe, since lasting barrier function of peritoneum after total peritonectomy allows a low percentage of the instilled chemotherapy to reach systemic circulation. [4] Published data on European paediatric cohorts treated with this approach usually derive from retrospective analysis and describe limited case histories [1, 5, 6]. Therefore more data collection is needed, to evaluate selection criteria, treatment efficacy and long term outcome.

**Materials and methods:**
This study was planned as a retrospective bicentric analysis of a cohort of paediatric patients who underwent CRS and HIPEC in case of peritoneal tumour dissemination between August 2011 and August 2023 either at the University Medical Centre of Hamburg-Eppendorf or at the Düsseldorf University Hospital. Young adults (=/> 18 years old at time of surgery) were also included, since current oncological protocols regarding considered malignancies are extended to patients up to 25 years old. [7] Data on demographics, diagnosis, applied (neo)adjuvant chemotherapy, surgical intervention, HIPEC technic, intra- and postoperative complications as well as outcome were collected via chart review. A mere descriptive statistical analysis was performed, due to the limited number of cases.

**Results:**
Twelve patients undergone CRS and HIPEC in case of peritoneal tumour dissemination were identified. Though, a total amount of fifteen procedures were carried out, since re-do HIPEC was performed in three cases. Median age at time of first intervention was 15,5 years, ranging from 19 months to 23,8 years. Male to female ratio comprises 1,4:1. 58% (7/12) were diagnosed with desmoplastic small round cell tumour; other treated malignancies include adrenal gland carcinoma (n=1), colonic adenocarcinoma (n=1), Ewing sarcoma (n=1), embryonal rhabdomyosarcoma (n=1) and PNET-like ovarian tumour (n=1). Indication to CRS and HIPEC was tumour relapse in 3/12 cases, partial or poor response to chemotherapy and deep regional hyperthermia in 6/12 cases and peritoneal dissemination at time of first diagnosis in 3/12 cases. All patients were declared eligible for the procedure by a multidisciplinary team of experts (internal tumour board); specifically there was never a trace of extra abdominal metastatic disease midst decision making. One day prior to surgery renal and hepatic function were assessed. Peritoneal cancer index (PCI) ranged between 4 and 16 (median value=9). Completeness of cytoreduction (CC), according to surgical and histopathological records, was classified as 0 in 80% and as 1 in 20% of procedures. During HIPEC heated chemotherapy was administered through 4 to 5 capillary drains in the peritoneal cavity for 60-90 minutes with an inflow-temperature varying between 41°C and 42°C. Chemotherapeutic drug dosage averaged 60-100mg/m2 for cisplatin (CDDP), 15-15,3

mg/m2 for doxorubicin (ADR) and 19,3 mg/m2 for mitomycin. In 13/15 procedures a combined CDDP-ADR protocol was applied. The only patient with peritoneal metastasis of colonic adenocarcinoma required combination of CDDP and mitomycin [4]. After CRS and HIPEC patients were observed in the intensive care unit (ICU) for at least 48 hours. Grad 3 and 4 complications occurred respectively after 12% of all procedures. There were no surgery-related deaths. After this procedure 11/12 patients received further treatments depending on the underlying malignancy. Hitherto follow-up showed that 7 patients are alive. Abdominal relapse after first CRS and HIPEC was diagnosed in 3 patients, mostly related to hepatic tumour spread.

**Conclusion:**
To the best of our knowledge we have been able to present the largest German case series of paediatric patients with peritoneal dissemination of solid abdominal tumours approached with CRS followed by HIPEC. Few major complications and no surgery related deaths have been registered, thus confirming this procedure as safe and feasible in selected cases. Accurate patient selection plays a key role in treatment related outcome and requires intensive cooperation with paediatric oncologists. Oncological expertise is necessary to establish the most appropriate HIPEC-protocol. From a mere surgical perspective, macroscopic resection radicality is mandatory to lower relapse rate and prolong disease free survival. Since experience in CRS and HIPEC concerning paediatric population is limited and heterogenous strategies are applied in different performing medical centres, more (prospective) trials with homogenised treatment guidelines are needed to identify eligible target patients, to gain reliable data on therapy efficacy as well as to assess optimal HIPEC protocols.

**References:**
[1] F. Bautista et al., 'Hyperthermic intraperitoneal chemotherapy after cytoreductive surgery for the treatment of peritoneal carcinomatosis in pediatric solid malignancies: a single institution experience', J. Pediatr. Surg., vol. 49, no. 8, pp. 1276–1279, Aug. 2014, doi: 10.1016/j.jpedsurg.2014.01.054.
[2] B. Rau, A. Königsrainer, F. Mohamed, and P. H. Sugarbaker, Eds., Peritoneal Tumors and Metastases: Surgical, intraperitoneal and systemic therapy. Cham: Springer International Publishing, 2021. doi: 10.1007/978-3-030-62640-2.
[3] A. Hayes-Jordan et al., 'Management of desmoplastic small round cell tumor', Semin. Pediatr. Surg., vol. 25, no. 5, pp. 299–304, Oct. 2016, doi: 10.1053/j.sempedsurg.2016.09.005.
[4] L. Sorrentino et al., 'Peritoneal carcinomatosis from colorectal cancer in the pediatric population: Cytoreductive surgery and HIPEC. A systematic review', Eur. J. Surg. Oncol., vol. 47, no. 2, pp. 211–215, Feb. 2021, doi: 10.1016/j.ejso.2020.08.021.
[5] J. Gesche et al., 'Cytoreductive surgery and hyperthermic intraperitoneal chemotherapy: A safe treatment option for intraperitoneal rhabdomyosarcoma in children below 5 years of age', Pediatr. Blood Cancer, vol. 66, no. 2, p. e27517, Feb. 2019, doi: 10.1002/pbc.27517.
[6] G. Seitz et al., 'Zytoreduktive Chirurgie und HIPEC bei peritoneal-metastasierten Tumoren im Kindesalter', Zentralblatt Für Chir. - Z. Für Allg. Visz. Thorax- Gefäßchirurgie, vol. 139, no. 06, pp. 607–612, Dec. 2014, doi: 10.1055/s-0034-1383240.
[7] R. D. Issels, R. Fietkau, and R. Wessalowski, 'Interdisziplinäre Arbeitsgruppe Hyperthermie (IAH)', Forum (Genova), vol. 37, no. 3, pp. 224–226, Jun. 2022, doi: 10.1007/s12312-022-01078-4.

Abstracts – DCK 2024 – 141st Congress of the German Society of Surgery – Leipzig, April 24–26 • DOI 10.1515/iss-2024-2005
Innov Surg Sci 2024; 9 (Special Suppl 1): s209–s252

**Cytoreductive surgery and hyperthermic intraperitoneal chemotherapy (HIPEC) in children – a case series of 5 patients undergoing 6 CRS-HIPEC procedures**
ID: 581

Mark Schneider[1 2], Ruben Visschers[1 2], Udo Kontny[3], Ulf Neumann[1 2], Wilhelmus van Gemert[1 2]

[1]*Department of General, Visceral and Transplant Surgery, University Hospital RWTH Aachen, Aachen, Deutschland*, [2]*Department of Surgery, UMC Maastricht, Maastricht*, [3]*Division of Pediatric Hematology, Oncology and Stem Cell Transplantation, , University Hospital RWTH Aachen, Aachen, Deutschland*

**Background:**
Cytoreductive Surgery (CRS) and hyperthermic intraperitoneal chemotherapy (HIPEC) is an effective treatment against peritoneal metastasis especially in adults but remains poorly documented in children. Our purpose is to present our institutional experience in performing complete cytoreductive surgery and HIPEC in children.

**Materials and methods:**
This retrospective single centre experience analysed all patients with peritoneal tumour spread treated by cytoreductive surgery (CRS) and HIPEC from 2011-2023 in University Hospital Aachen.

**Results:**
Five patients (1 female, 4 male) underwent six CRS-HIPEC procedures from 2011 until 2023. The mean age at admittance to hospital was 10.6 years. Two patients suffered from desmoplastic small round cell tumours, the other three from hepatocellular carcinoma with intraabdominal spread, peri-testicular rhabdomyosarcoma with an abdominal recurrency and recurrent nephroblastoma, respectively. In all patients CC-0 resection status was achieved. Median hospital stay was 10 days after CRS and HIPEC with minor complications. Median length of hospital stay was 10 (range: 7 - 23) days with low complications. Two patients were alive at the last follow up. 4 patients developed a recurrent disease. One patient with desmoplastic small round cell tumour was treated with adjuvant radiotherapy and developed 3 liver metastases successfully treated with radio frequency ablation and Irreversible Electroporation (IRE). A cerebral metastasis was resected. Another patient with desmoplastic small round cell tumour stayed disease free and is still alive 8 years after CRS and HIPEC. The patient with nephroblastoma developed non-resectable recurrent abdominal tumours and was treated with palliative intention. In case of a recurrent HCC secondary CRS and HIPEC procedure can be performed. 17 months later a resection of recurrent abdominal tumour and CRS alone was performed. A new hepatic lesion was treated with radio frequency ablation 6 years after initial HIPEC and CRS. In this case, a survival of nearly 10 years after the first HIPEC procedure was achieved.

**Conclusion:**
Cytoreductive Surgery and HIPEC can be performed safely, with few complications. Furthermore, it can result in long-term disease-free survival, in otherwise lethal diseases.

**Synergistic Anti-Tumor Growth Effect of Hypericin Combined with Simvastatin in Alveolar Rhabdomyosarcoma: In Vitro Study**
ID: 717

Lajwanti Dhaka[1], Faulthe Theresa[1], Marie Woll[1], Mohammad Vahdad[1], Illya Martynov[1], Guido Seitz[1]

[1]*Department of Pediatric Surgery and Urology, University Hospital Giessen-Marburg*

**Background:**
Rhabdomyosarcoma (RMS) is highly aggressive soft-tissue cancer that primarily affects children and young adults. The prognosis for patients with metastatic alveolar RMS remains largely unsatisfactory despite the adoption of intensive multimodal therapy. Thus, development of new therapeutic approaches is essential to improve patient´s outcomes. Photodynamic therapy (PDT) is an emerging new treatment approach being explored for a variety of malignancies. It involves the administration of a photosensitiser such as hypericin (HYP), which targets the tumor cells, either topically or systemically. However, PDT has limitations in terms of tumor depth penetration and may not be therefore suitable for deep-seated tumors. We aimed to augment the efficacy of PDT/HYP by combining it with Simvastatin, a cholesterol-lowering medication that has strong anticancer effects.

**Materials and methods:**
The RH-30 cells were subjected to various treatments including: 1) HYP alone, 2) Simvastatin alone, 3) HYP/Simvastatin, 4) PDT/HYP, and 5) PDT/HYP/Simvastatin and a control group. The readout parameters included cell viability and apoptosis rates. The apoptotic cell population was identified with the Propidium iodide dye and the early apoptotic cells with the PE Annexin V stain. Furthermore, a wound healing assay was performed to evaluate the effects of these treatments on the rate of cell migration.

**Results:**
Treatment of RH-30 cells with HYP alone and Simvastatin alone resulted in a cell viability of 62% ($p=0.37$) and 59% ($p=0.03$) compared to control group, respectively. After combination of HYP with Simvastatin the cell viability decreased to 21% ($p=0.01$). When HYP was administered in combination with PDT, cell viability decreased to 17% ($p=0.02$). The combination of HYP + Simvastatin + PDT resulted in a further reduction in cell viability to 13% ($p=0.0002$). In the apoptotic assay, treatment with HYP alone and Simvastatin alone resulted in 13% and 14% apoptotic cells, respectively. The combination of HYP/Symvastatin increased the effect to 49.5%, suggesting a synergistic effect. PDT further enhanced cell apoptosis, resulting in 89% and 96%, respectively. In wound healing assay, the combined treatment of Simvastatin and HYP showed significant reduction of wound healing within 24 hours.

**Conclusion:**
Results of our study indicate that HYP enhances the cytotoxic effects of Simvastatin on alveolar RMS *in vitro*. This drug combination has a potential for testing in preclinical *in vivo* studies.

Abstracts – DCK 2024 – 141st Congress of the German Society of Surgery – Leipzig, April 24–26 • DOI 10.1515/iss-2024-2005
Innov Surg Sci 2024; 9 (Special Suppl 1): s209–s252

**Lung Function, Cardiorespiratory Endurance and Health-Related Quality of Life after Operative Therapy versus Watch-and-wait in Asymptomatic Congenital Malformations of the Lung**
ID: 189

Richard Martel[1], Julia Elrod[1], Thomas Schaible[2], Michaela Klinke Petrowsky[1], Meike Weis[3], Michael Boettcher[1]

[1]*Universitätsklinikum Mannheim, Klinik für Kinder- und Jugendchirurgie, Mannheim, Deutschland*, [2]*Klinik für Neonatologie, Universitätsklinikum Mannheim, Mannheim, Deutschland*, [3]*Klinik für Radiologie und Nuklearmedizin, Universitätsklinikum Mannheim, Mannheim, Deutschland*

**Background:**
Operative therapy vs. watch and wait remains a debated topic in the management of asymptomatic congenital malformations of the lung, i.e. Congenital Pulmonary Airway Malformation (CPAM) and pulmonary sequestration. While operative treatment is increasingly recommended by many institutions, a differentiated approach according to the type of the malformation and presence of genetic risk factors have been developed recently for CPAM. However, diagnostic uncertainty in terms of classification limits the application of the watch and wait strategy. To add arguments to the discussion of these approaches, the aim of our study was to compare the outcome between non-operative management and resection in cases of asymptomatic lung malformations with respect to infections, lung function and motility of the diaphragm, physical fitness and health-related quality of life.

**Materials and methods:**
A cross-sectional analysis was carried out throughout our cohort of almost 200 children that were treated in our center within the last 15 years with CPAM or sequestration. We assessed the history of infections and examined diaphragmatic motility using ultrasound, cardiorespiratory endurance by the shuttle run test as well as spirometry parameters in 60 patients, health-related quality of life was measured using validated questionnaires (HrQoL) in 40 patients. Patients were classified into four groups: non-operatively management, primary resection, secondary operation due to infections and secondary operation due to progress of the CPAM or sequestration. The data was analyzed age-adjusted and stratified by the extent of resection, the volume and classification of the malformation in the neonatal period. Excluded were children that were prenatally treated by ethanol ablation of the CPAM.

**Results:**
Our preliminary data presents differences in fitness, diaphragmatic motility and spirometry data but no differences in most aspect of HrQoL between the groups. Further analysis will be performed.

**Conclusion:**
It appears that early resection of lung malformation may be beneficial. Further analysis our data may reveal risk factors for failure of non-operative treatment.

**Short Bar Technique for Pectus Excavatum – Single Institutional Experience**
ID: 385

Peter Zimmermann[1], Sebastian Krämer[2], Daniel Gräfe[3], Franz Wolfgang Hirsch[3], Martin Lacher[1]

[1]*Universitätsklinikum Leipzig, Klinik und Poliklinik für Kinderchirurgie, Leipzig, Deutschland*, [2]*Universitätsklinikum Leipzig, Klinik und Poliklinik für Viszeral-, Transplantations-, Thorax- und Gefäßchirurgie, Leipzig, Deutschland*, [3]*Universitätsklinikum Leipzig, Institut für Kinderradiologie, Leipzig, Deutschland*

**Background:**
Pectus excavatum (PE) is the most common chest wall deformity. After introduction of the minimally invasive repair of pectus excavatum (MIRPE) in 1998 by Donald Nuss, a modification, the so called “short bar technique“ (SBT) was developed by Hans Pilegaard in 2001. The latter utilizes shorter asymmetric bars and no additional fixing sutures to the chest wall.

**Materials and methods:**
From 8/2019 to 8/2023 (48 months) all adolescents and young adults (AYA) undergoing MIRPE using the SBT were included. Preoperative Haller index and Correction index were assessed using real-time MRI in the end-expiratory phase of quiet expiration. All cases were performed under general anesthesia with epidural analgesia. All patients were operated on by the same team of three senior surgeons. The number of bars needed to correct the malformation, the bar length, operating time, and complications were assessed.

**Results:**
Forty-two patients with PE were included (male-female ratio: 6:1; median age: 17 years (range 13-32 years); 25 patients (60%) < 18 years). The median Haller Index was 6.2 (range 2.9-16), and the median Correction Index was 35% (range 14-78%). The number of bars needed for correction was one in 22 patients (52%), two in 19 children (45%) and three in one patient (2%). The most frequently used bar length was 10 inches [N=37 (59%)] followed by 11 Inch [N=17 (27%)], 9 Inch [N=6 (10%)], 12 Inch [N=2 (3%)] and 13 Inch [N=1 (2%)]. The median operating time was 75 minutes (range 35-199 minutes). Patients were discharged after a median time of 5 days (range 3-13 days). Overall, 7 patients (17%) experienced surgical complications, classified by the Clavien-Dindo grading system: one seroma with needle drainage (grade IIIa), three pleural effusions with chest tube insertion (grade IIIa), one pneumothorax with chest tube insertion (grade IIIa), one deep wound infection with VAC therapy (grade IIIb), one dislocation of a stabilizer (grade IIIb). There were no grade IV/V complications. Bar removal was performed in two patients after 3 years.

**Conclusion:**
Although we cannot report on long-term results, we consider the SBT a straightforward procedure for correcting PE. Compared to the standard Nuss repair the main advantage is the stable bar position without the need for additional fixation. Moreover, the insertion of the bar is easier because of the short distance between the skin incisions as well as the entrance and exit points of the chest. However, long-term follow-up is needed to finally assess the benefit of the SBT compared to the traditional technique.

**Ante situ tumour resection with kidney autotransplantation for nephroblastoma and neuroblastoma in childhood**
ID: 146

Benjamin Mayer[1], Matthias Schunn[1], Lea-Sophie Weinpert[1], Ioan Cristian Urla[1], Jürgen Schäfer[2], Frank Fideler[3], Felix Neunhoeffer[4], Silvio Nadalin[5], Steven Warmann[1], Jörg Fuchs[1]

[1]*Universitätsklinikum Tübingen, Abteilung für Kinderchirurgie und Kinderurologie, Tübingen, Deutschland*, [2]*Radiologische Universitätsklinik Tübingen, Sektion Kinderradiologie, Tübingen, Deutschland*, [3]*Universitätsklinik für Anästhesie und Intensivmedizin, Sektion Kinderanästhesie, Tübingen, Deutschland*, [4]*Universitätsklinikum Tübingen - Klinik für Kinderheilkunde und Jugendmedizin, Abteilung für Kinderkardiologie, Pulmologie und Intensivmedizin, Tübingen, Deutschland*, [5]*Universitätsklinikum Tübingen, Abteilung für Allgemeine-, Viszeral- und Transplantationschirurgie, Tübingen, Deutschland*

**Background:**
In childhood nephroblastoma and distinct conditions of neuroblastoma, complete tumor resection is the primary surgical goal. In cases of renal involvement, nephron-sparing surgery (NSS) might become necessary with the major aim to preserve as much renal tissue as possible. Eventually, the risk of loss of renal function over time needs to be reduced, which potentially leads to the necessity of dialysis. The aim of this study was to evaluate the role of ante situ resection with renal autotransplantation in children suffering either from bilateral nephroblastoma or from complex midline neuroblastoma involving the kidneys.

**Materials and methods:**
A retrospective single-center cohort study of consecutive children with bilateral nephroblastoma and complex midline neuroblastoma undergoing renal autotransplantation at our institution was performed. All children had undergone neoadjuvant chemotherapy. The diagnostic work-up consisted of cross-sectional imaging and was supplemented by intraoperative ultrasound. Indication for renal autotransplantation was established intraoperatively, when the tumor resection was supposedly too complex and time consuming to be executed under in situ vascular exclusion. Autotransplantation was performed in cooperation with a transplant surgeon from our center. Renal arteries and veins were transected and the kidneys were perfused ante situ with transplant rinsing solution. After tumor resection, the vessels were re-anastomosed at their original sites.

**Results:**
Between 2006 and 2023, 2 children (1 girl, 1 boy) with bilateral nephroblastoma and 3 children with midline neuroblastoma (2 girls, 1 boy) underwent renal autotransplantation during tumor resection. The median age at surgery was 40 months (range 20-62) and the median operative time was 450 minutes (range 389-590). Complete tumor resection was achieved in 3 patients (2 with nephroblastoma, 1 with neuroblastoma) and gross-total tumor resection of more than 95% of the visible tumor in 2 patients with neuroblastoma. Sepsis led to death in 1 patient with high-risk neuroblastoma 14 days after gross total tumor resection. Oncological outcome at a median follow-up of 32 months (range 9 - 387) was no evidence of disease in all 3 patients after complete tumor resection and local control in 1 patient with neuroblastoma after gross total tumor resection. Glomerular filtration rates were normal in all children, 2 children required antihypertensive treatment.

**Conclusion:**
Ante situ tumor resection with renal autotransplantation represents a highly complex surgical procedure, which can be performed with sufficient oncologic and functional outcome in children with bilateral nephroblastoma for whom in situ nephron-sparing surgery is not possible. It is also a possible approach in children with neuroblastoma, in which complete tumor resection is necessary but not otherwise possible. This approach contributes to reducing the risk of loss of renal function after tumor resection, which potentially leads to the necessity of dialysis.

**First results after antegrade ESIN for unstable diametaphyseal forearm fractures in the pediatric age group**
ID: 15

Markus Dietzel[1], Simon Scherer[1], Jakob Spogis[2], Hans Joachim Kirschner[1], Jörg Fuchs[1], Justus Lieber[1]

[1]*Universitätsklinikum Tübingen, Kinderchirurgie und Kinderurologie, Tübingen, Deutschland*,
[2]*Universitätsklinikum Tübingen, Kinderradiologie, Tübingen, Deutschland*

**Background:**
Treatment of unstable forearm fractures in the diametaphyseal transition zone is still a matter of debate. Alternatives advocated for this difficult region include classic techniques of percutaneous Kirschner wire (K-wire) fixation and elastic stable intramedullary nailing (ESIN) as well as plate osteosynthesis, modified techniques such as transepiphyseal intramedullary K-wire fixation, and others [1, 2]. A procedure in which an elastic nail was inserted antegrade, dorsally, in the middle third of the shaft of the radius, had long been described in adults [3]. This lead to the development of antegrade ESIN in children which now is a promising treatment method including the advantages of the classic ESIN technique. As for complications, potential injury to the radial nerve has been described. This study reports first results of a consecutive patient cohort after antegrade ESIN in the above-mentioned injury and patient context.

**Materials and methods:**
The diametaphysis of the forearm was defined as the square over the joints of both forearm bones subtracted with the square over the metaphysis of the radius alone [4]. The data of all patients <16 years of age who received antegrade ESIN for an unstable diametaphyseal forearm fracture at a single high-volume pediatric trauma center were retrospectively analyzed. Insertion of the intramedullary nail was performed using a modified anterolateral approach according to the description of Thompson [5]. This procedure is realized using an incision measuring no longer than 2 cm which is located on the center of a line connecting the lateral epicondyle of the humerus to the dorsoradial tubercle (Lister's tubercle). A “soft spot” between the extensor carpi radialis muscles and the group of extensors of the thumb and fingers provides access to the dorsal aspect of the radial shaft, avoiding injuries of essential nerves or vessels.

**Results:**
Between 01/2022 and 09/2023 ten patients were treated using antegrade ESIN of the radius. The mean age was 10.2 years (6–15 years); the gender distribution was 5:5 boys/girls; the right forearm was more frequently affected (7:3). A concomitant distal ulna fracture was documented in 9/10 patients of which 6 received traditional antegrade ESIN of the ulna in order to gain sufficient stability. Additional cast immobilization was performed in 3/10 patients for 28 days. Fractures progressed to full union in axial alignment in 8/10 cases. In 1 case, temporary motor-sensory malfunction occurred for a few postoperative days, but nerve sonography excluded structural injury. An inaccurate choice of the implant site (the above-mentioned “soft spot”) as a possible cause was discussed. This patient also suffered a refracture two months after the initial treatment leading to closed reduction and re-bending of the implant under general anesthesia. In 1 case, secondary dislocation (9° ulnar deviation of the distal radius in the frontal plane) was seen on plain X-ray at the timepoint of consolidation, but range of motion was not impaired. Metal removal was performed after an average of 145 days (119–168 days) under general anesthesia in a day-surgery setting.

**Conclusion:**
Antegrade ESIN in pediatric diametaphyseal forearm fractures is minimally invasive, quick and technically easy, and proved to be safe in order to achieve full recovery of forearm rotation without cosmetic deformity. Respecting the anatomical landmarks of the dorsal forearm makes injury to the radial nerve unlikely. This technique, performed at the radius alone or combined with ESIN of the ulna

Abstracts – DCK 2024 – 141st Congress of the German Society of Surgery – Leipzig, April 24–26 • DOI 10.1515/iss-2024-2005
Innov Surg Sci 2024; 9 (Special Suppl 1): s209–s252

in both bone fractures, potentially offers cast-free postoperative care and early spontaneous mobilization. To support these findings, further data should be collected prospectively and in a randomized and registry-based manner.

References:

[1] Lieber J et al. Eur J Ped Surg 2010
[2] Kubiak R et al. Medicine 2019
[3] Sato O et al. J Hand Surg Am 2002
[4] Lieber J et al. Unfallchirurg 2011
[5] Thompson JE. Ann Surg 1918

Abstracts – DCK 2024 – 141st Congress of the German Society of Surgery – Leipzig, April 24–26 • DOI 10.1515/iss-2024-2005
Innov Surg Sci 2024; 9 (Special Suppl 1): s209–s252

**Inguinal Handlebar Injury in a Pediatric Patient: A Near Miss from Potential Limb Loss**
ID: 768

Hesham Elsayed[1], Georg Singer[1], Agnes Bokros[1], Elena Friehs[1], Saskia Hankel[1], Claus-Uwe Weitzer[1], Thomas Petnehazy[1], Martin Münnich[1], Ferdinand Füsi[1], Holger Till[1]

[1]*Medizinische Universität Graz, Univ.-Klinik für Kinder- und Jugendchirurgie, Graz, Österreich*

**Background:**
Handlebar injuries that do not penetrate may still have devastating intraabdominal such as intestinal injury, solid organ injury, or genitourinary injury. Management guidelines typically advise for multimodal radiological assessments and mainly conservative treatment. Hollow organ perforations or cardio-pulmonary instability, however, require surgical interventions. But what about inguinal handlebar injuries? Can they also cause devastating injuries?

**Materials and methods:**
In this report, we present the case of a ten-year-old boy who sought medical attention after a bicycle accident, resulting in an injury to the external abdominal muscle in the left iliac region.

**Results:**
A ten-year-old male patient presented with localized abdominal pain, tenderness, and swelling in the lower abdominal quadrant, extending into the left scrotal compartment after a bicycle accident. Thorough clinical examination and imaging studies, including ultrasound and computed tomography, confirmed the diagnosis of abdominal wall injury. Remarkably, the damaged contents were limited to the aponeurosis of the external abdominal muscle and a notable lesion of the epigastric vessels. The trauma was extensive, exposing the iliac vessels but without involvement. Despite the patient's stable condition, intensive close monitoring was carried out before a carefully planned surgical exploration was conducted. Laparoscopic exploration revealed an intact peritoneum. External exploration revealed a complete tear of the aponeurosis of the external abdominal muscle, a significant hematoma due to the epigastric artery lesion, but no involvement of the iliac vessels. The patient underwent evacuation of the hematoma, extensive lavage of the defect and primary closure of the abdominal wall defect. The postoperative recovery was uneventful, and after five weeks, the patient could return to normal daily activities.

**Conclusion:**
This case highlights the diverse clinical presentations of handlebar injuries in children and emphasizes the importance of tailored management strategies based on the specific anatomical involvement. Early recognition, accurate diagnosis, and prompt surgical intervention, considering the individual characteristics of the trauma, are crucial for achieving favorable outcomes. However, clear recommendations for handlebar traumas are currently lacking. Further research and data collection are necessary to establish evidence-based guidelines for the management of this rare condition.
